# Inhibition of Hedgehog signaling suppresses proliferation and microcyst formation of human Autosomal Dominant Polycystic Kidney Disease cells

**DOI:** 10.1038/s41598-018-23341-2

**Published:** 2018-03-21

**Authors:** Luciane M. Silva, Damon T. Jacobs, Bailey A. Allard, Timothy A. Fields, Madhulika Sharma, Darren P. Wallace, Pamela V. Tran

**Affiliations:** 1 Department of Anatomy and Cell Biology, University of Kansas Medical Center, Kansas City, KS USA; 2 Department of Pathology and Laboratory Medicine, University of Kansas Medical Center, Kansas City, KS USA; 3 Department of Molecular and Integrative Physiology, University of Kansas Medical Center, Kansas City, KS USA; 4 Department of Internal Medicine, University of Kansas Medical Center, Kansas City, KS USA; 5 Jared Grantham Kidney Institute, University of Kansas Medical Center, Kansas City, KS USA

## Abstract

Autosomal Dominant Polycystic Kidney Disease (ADPKD) is caused by mutation of *PKD1* or *PKD2*, which encode polycystin 1 and 2, respectively. The polycystins localize to primary cilia and the functional loss of the polycystin complex leads to the formation and progressive growth of fluid-filled cysts in the kidney. The pathogenesis of ADPKD is complex and molecular mechanisms connecting ciliary dysfunction to renal cystogenesis are unclear. Primary cilia mediate Hedgehog signaling, which modulates cell proliferation and differentiation in a tissue-dependent manner. Previously, we showed that Hedgehog signaling was increased in cystic kidneys of several PKD mouse models and that Hedgehog inhibition prevented cyst formation in embryonic PKD mouse kidneys treated with cAMP. Here, we show that in human ADPKD tissue, Hedgehog target and activator, Glioma 1, was elevated and localized to cyst-lining epithelial cells and to interstitial cells, suggesting increased autocrine and paracrine Hedgehog signaling in ADPKD, respectively. Further, Hedgehog inhibitors reduced basal and cAMP-induced proliferation of ADPKD cells and cyst formation *in vitro*. These data suggest that Hedgehog signaling is increased in human ADPKD and that suppression of Hedgehog signaling can counter cellular processes that promote cyst growth *in vitro*.

## Introduction

Autosomal Dominant Polycystic Kidney Disease (ADPKD) is among the most commonly inherited, life-threatening diseases, affecting 1:500 adults worldwide. ADPKD is characterized by the formation and growth of fluid-filled cysts in the kidneys, which compress neighboring tubules, resulting in renal injury and fibrosis. Many of these patients progress to end stage renal disease (ESRD) by the 6^th^ decade of life. The molecular mechanisms underlying ADPKD are complex, involving misregulation of multiple signaling pathways and aberration of multiple cellular processes, including increased cell proliferation, fluid secretion, apoptosis and incomplete differentiation of tubular epithelial cells^1^. Most ADPKD cases result from mutations in *PKD1* or *PKD2*, which encode polycystin-1 (PC1) and polycystin-2 (PC2) transmembrane proteins, respectively. PC1 and PC2 localize to the primary cilium, a non-motile sensory organelle, and form a functional complex that is thought to mediate signaling pathways^2,3^.

Mutation of most ciliary genes causes renal cystic disease^4^; however, the role of ciliary dysfunction in renal cystogenesis remains unclear. Paradoxically, genetic ablation of primary cilia in *Pkd1* and *Pkd2* conditional knock-out mice attenuated PC-mediated renal cystogenesis, which led to the proposal that an undefined cilia-dependent signaling pathway promotes PC*-*deficient cyst formation^5^. Consistent with these data, pharmacological shortening of primary cilia in *Nek8*^*jck/jck*^ mouse mutants, which model ADPKD, ameliorated *Nek8*^*jck/jck*^ renal cystic disease^6^.

The Hedgehog (Hh) signaling pathway is among the best characterized ciliary-mediated pathways. Hh signaling controls cell proliferation, differentiation and cell fate, and is essential for development and tissue homeostasis^7^. In the canonical pathway, binding of Hh ligand to the Patched (PTCH1) receptor at the cilium promotes ciliary exit of PTCH1 and ciliary entry and activation of the Smoothened (SMO) signal transducer^8,9^. The signal is transduced ultimately to the Glioma (GLI) transcriptional factors and final mediators of the pathway, whose activity is also regulated at the cilium.

Cilia are formed by intraflagellar transport (IFT), the bi-directional transport of protein cargo along the ciliary axoneme by IFT-B and -A complexes. In mice, loss of most IFT-B proteins causes absent or stunted cilia and the inability to respond to the Hh signal^10^. In contrast, loss of the IFT-A proteins, THM1 (TTC21B) and IFT122, results in accumulation of proteins in bulb-like structures at the distal tip of shortened cilia and enhanced activation of the Hh pathway^11,12^. Deletion of *Ift-B* or *-A* genes in the kidney or globally during late embryogenesis causes renal cysts^13–15^.

Hh signaling has been reported to promote renal proliferative diseases, including renal cell carcinoma^16,17^ and fibrosis^18^, and several studies suggest Hh signaling may also influence cystogenesis^19–22^. Cystic kidneys of several mouse models have shown upregulation of *Gli1*, a transcriptional target of the Hh pathway^14,21–23^, and Hh inhibition reduced cAMP-mediated cysts of cultured embryonic kidneys of several PKD mouse models^22^. Further, a transcriptome analysis of human ADPKD kidneys revealed increased expression of Hh signaling components^24^. Thus, we sought to extend our analyses of Hh signaling in renal cystogenesis to human ADPKD. To this end, we examined Hh status in human ADPKD renal tissue and primary cystic epithelial cells and assessed the effect of Hh modulators on ADPKD cell proliferation and cyst formation *in vitro*.

## Results

### GLI1 is upregulated in human ADPKD renal tissue

**Figure 1:**
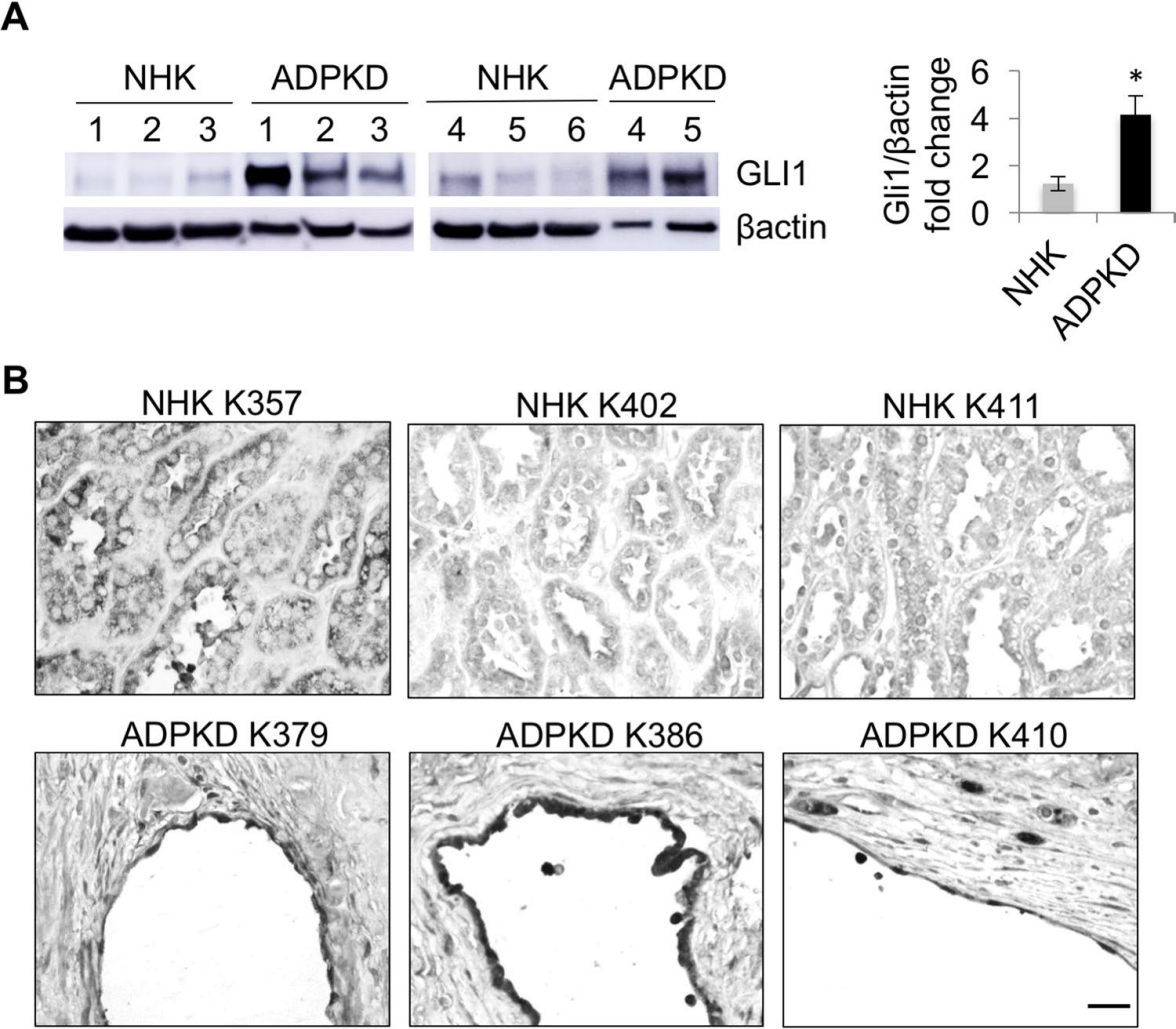
GLI1 is upregulated in human ADPKD renal tissue. (**A**) Western blot analysis for GLI1 in normal human kidney (NHK) and ADPKD extracts of the renal cortex. Bars (mean ± SEM) are band intensity normalized to β-actin, and represented as fold change from NHK, set to 1.0. Quantification of GLI1 levels was performed on 6 NHK and 5 ADPKD tissue extracts (Summary Table S1). Statistical significance was determined by an unpaired t-test. *P < 0.05 (**B**) Immunohistochemistry for GLI1 on NHK and ADPKD sections of the renal cortex. Scale bar = 50 μm.

**Figure 2:**
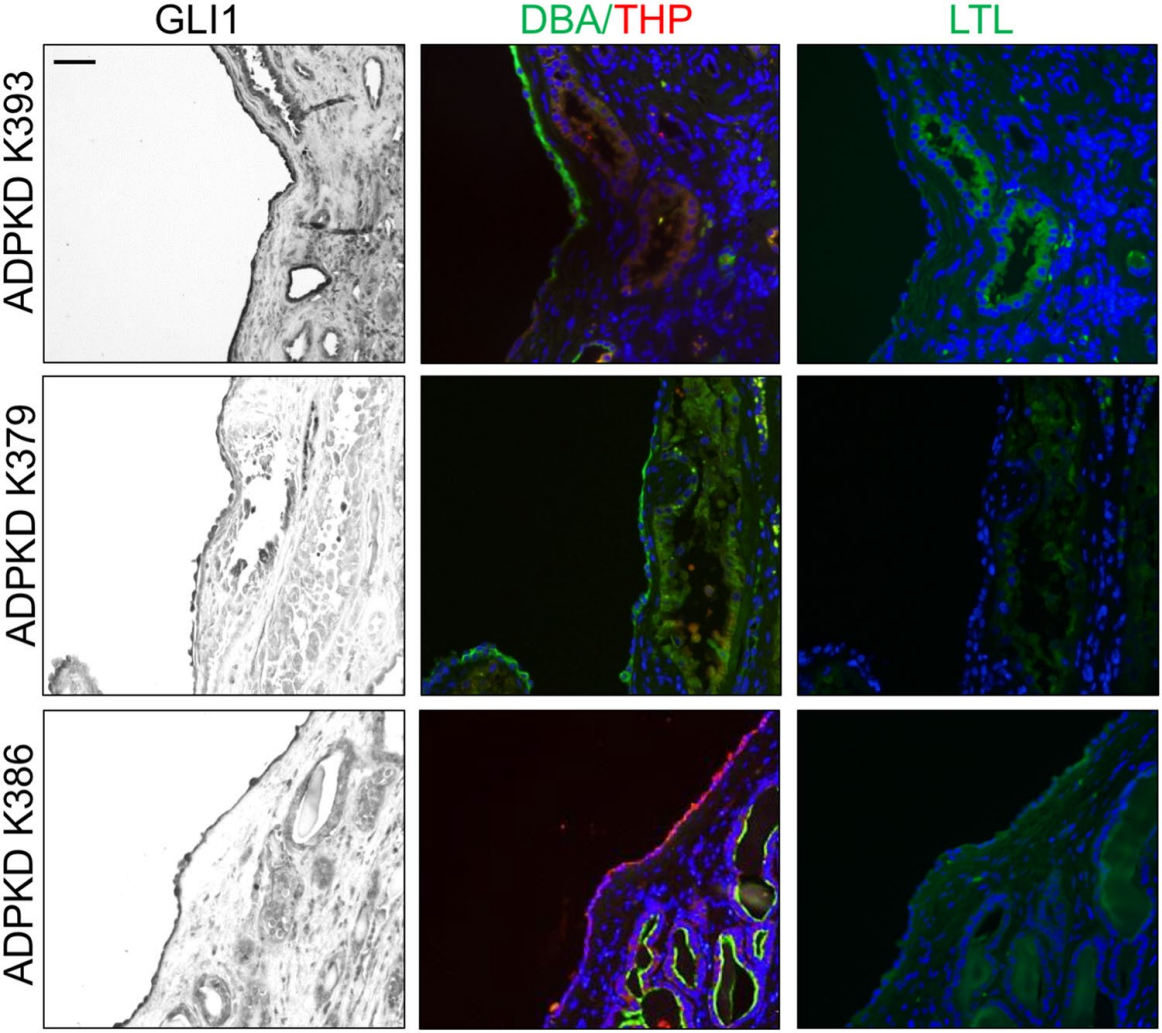
GLI1-expressing epithelial cells derive from collecting duct and Loop of Henle tubules. GLI1 immunohistochemistry and staining with DBA, LTL and THP on ADPKD sections of the renal cortex. Scale bar = 100 μm.

We performed Western blot analysis for GLI1, a target and activator of the Hh pathway. We found increased GLI1 in ADPKD compared to normal human kidney (NHK) tissue (Fig. 1A; Figures S1; S2). Using immunohistochemistry, we observed more intense nuclear GLI1 staining in interstitial cells and in epithelial cells lining some cysts of ADPKD tissue (Fig. 1B; Figure S3). We next incubated ADPKD tissue sections with fluorescein-conjugated *Lotus tetragonolobulus* (LTL) or *Dolichos biflorus* agglutinin (DBA) lectins or with antibody against Tamm-Horsfall Protein (THP) to examine the tubular origin of GLI1 + cells. While cystic cells did not label with LTL, a marker of proximal tubules, DBA or THP staining of cystic cells suggested that the cysts originated from collecting duct or Loop of Henle tubules, respectively, and that GLI1-positive epithelial cells were present in these cysts (Fig. 2; Figure S4).

### Ciliary trafficking and Hedgehog signaling are intact in ADPKD primary renal epithelial cells

**Figure 3:**
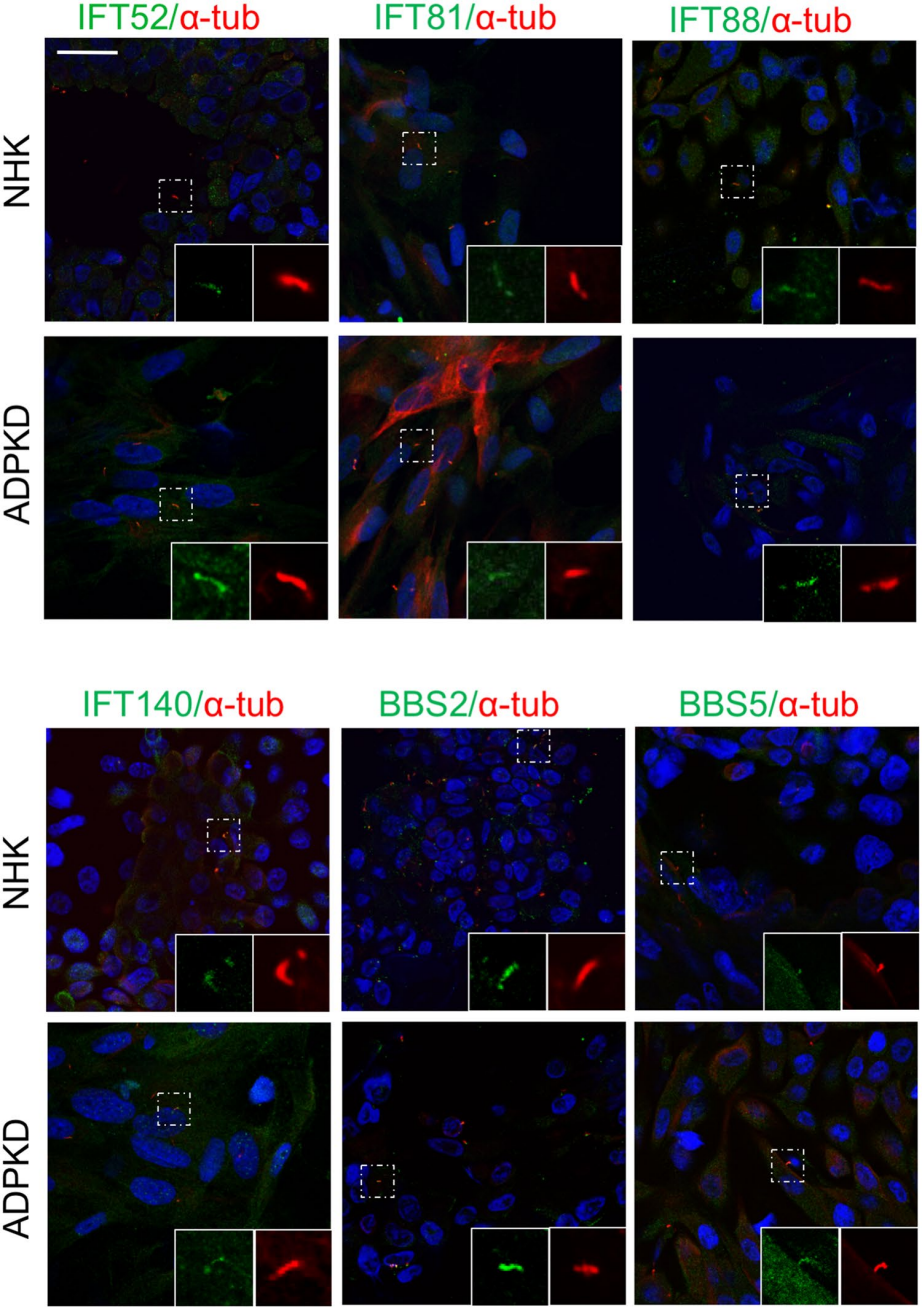
Primary cilia of ADPKD cells show normal localization of IFT and BBS proteins. Immunofluorescence for IFT52 (green), IFT81 (green), IFT88 (green), IFT140 (green), BBS2 (green), and BBS5 (green) and acetylated α-tubulin (red) in NHK and ADPKD cells. Scale bar = 25 μm. Localization of each ciliary protein was examined in a minimum of 3 NHK and 3 ADPKD cell lines (Summary Table S1).

In mice, ciliary length appears to affect PKD severity^5,6^. Further, increased ciliary length has been reported in the *Pkd1*^*RC/RC*^ mutant mouse, which harbors an ADPKD mutation^25^, and in *Pkd1*^*−/−*^ and *Pkd2*^*−/−*^derived embryonic renal epithelial cells^26^. Since ciliary length can be modified via IFT, and IFT affects Hh signaling^10^, we examined ciliary localization of IFT components in primary epithelial cells derived from the cortex of NHK kidneys or from surface cysts of ADPKD kidneys. The majority of NHK and ADPKD primary renal epithelial cells stained with varying intensities for DBA, but not for LTL or THP (Figure S5), suggesting most cells originate from collecting ducts, consistent with previous reports^27,28^. In ADPKD primary cells, IFT-B components, IFT52, IFT81 and IFT88, and IFT-A component, IFT140, localized throughout the cilium, similar to their localization in NHK cells (Fig. 3).

The BBSome is an 8-unit protein complex that shuttles protein cargo to cellular membranes and throughout the ciliary membrane and has been suggested to regulate the ciliary import and export of PC1 and PC2. *Bbs1* knock-down or expression of a dominant-negative form of Bbs3 in IMCD cells resulted in absence of PC1 in the cilium^29^, while combined deficiency of *BBS4* and *BBS5* in retinal pigment epithelial (RPE) cells caused ciliary accumulation of PC2^30^. To determine if the BBSome is conversely affected in ADPKD, we examined the localization of BBS components, BBS2 and BBS5 (Fig. 3). Similar to the IFT proteins, the BBS proteins localized normally along the ciliary axoneme. Together, these data suggest that polycystin dysfunction does not overtly affect the ciliary trafficking machinery.

**Figure 4:**
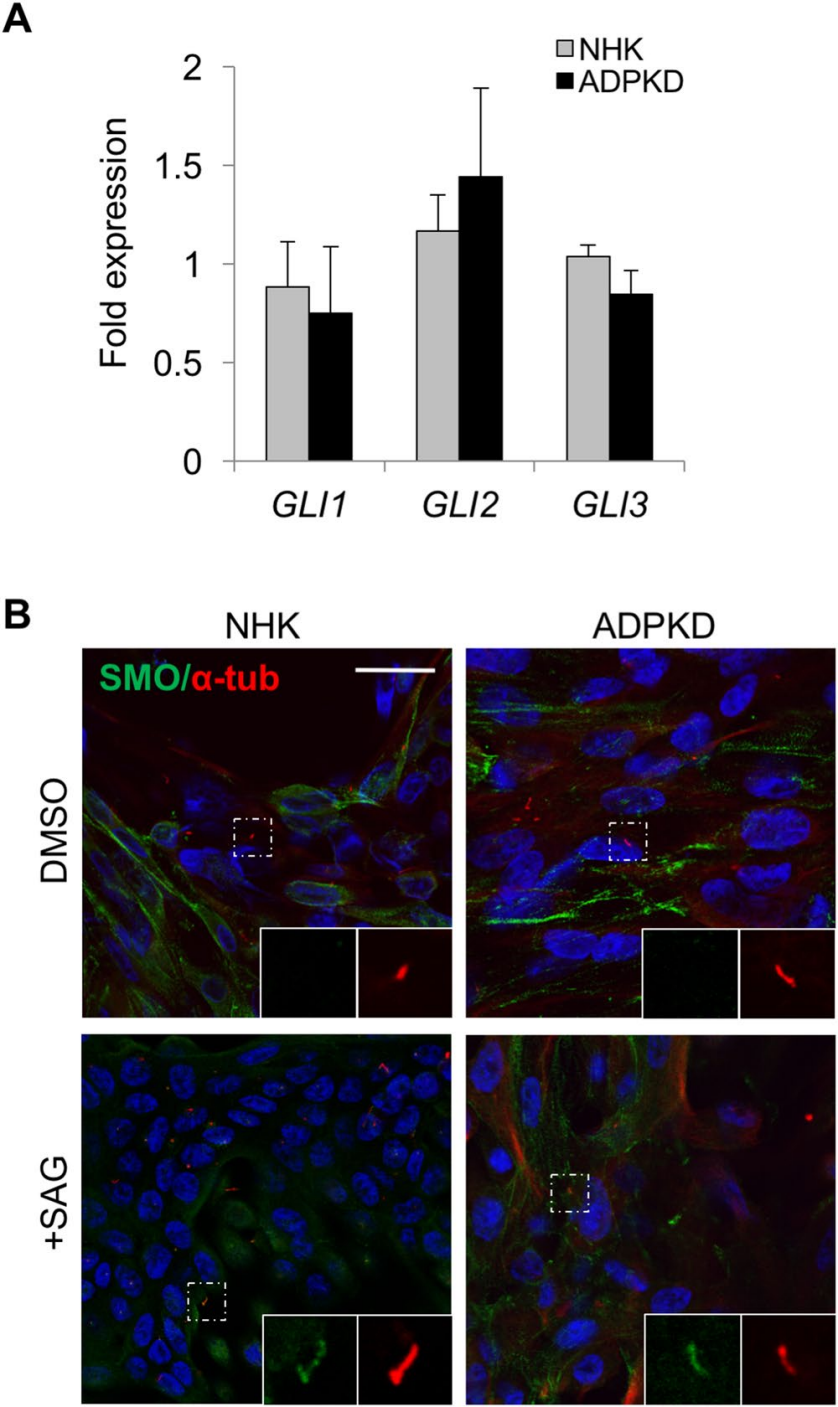
Human primary renal epithelial cells have Hh signaling machinery. (**A**) qPCR analysis on NHK and ADPKD primary renal epithelial cells. Bars represent mean ± SEM of 3 NHK and 3 ADPKD cell lines (Summary Table S1). (**B**) Immunofluorescence for SMO (green) and acetylated α-tubulin (red) in presence or absence of SAG. Experiments were replicated in 5 NHK and 5 ADPKD cell lines (Summary Table S1). Scale bar = 25 μm.

We examined Hh status in ADPKD primary renal epithelial cells. Using qPCR, we found that *GLI1*, *GLI2* and *GLI3* transcript levels were similar in NHK and ADPKD cells (Fig. 4A). Additionally, we examined SMO localization, which enriches in the cilium upon pathway stimulation^8^. In the absence of Hh agonist, SMO was mostly undetected in primary cilia of NHK and ADPKD cells, but following treatment with SAG, a SMO agonist, NHK and ADPKD cells showed similar ciliary enrichment of SMO (Fig. 4B), suggesting similar Hh signaling levels. These data indicate that ADPKD primary renal epithelial cells have Hh signaling machinery and respond appropriately to Hh modulation.

### Hh inhibitors reduce cAMP-induced proliferation and microcyst formation of human primary ADPKD renal cells

**Figure 5:**
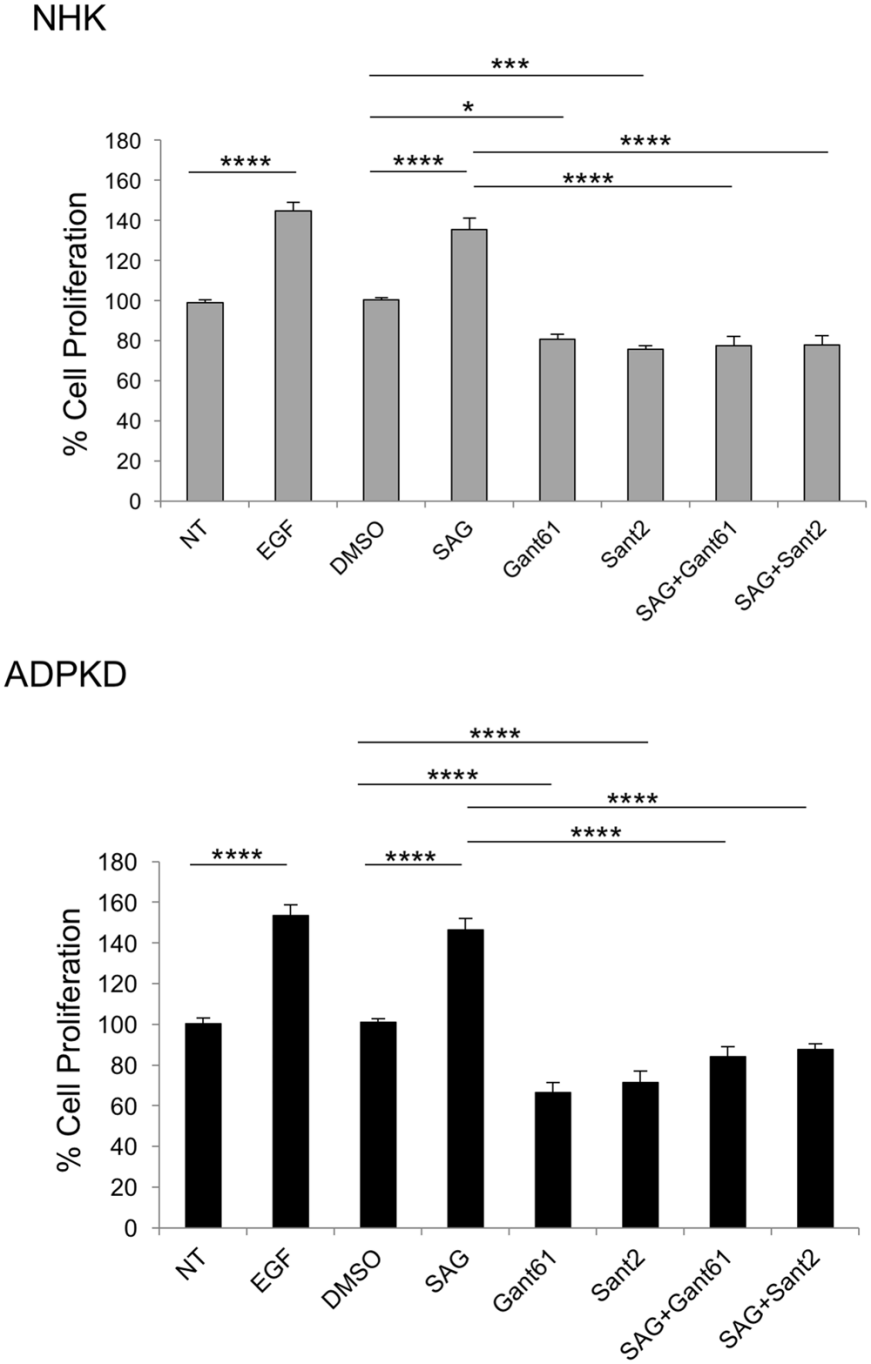
Hh inhibitors suppress proliferation of human primary renal epithelial cells. Percent cell proliferation of NHK and ADPKD cells. Cell proliferation was calculated as a proportion relative to cell number of the NT (no treatment) group, which was set at 100. NT-no treatment; EGF – epidermal growth factor; SAG – Smoothened agonist; SANT2- Smoothened antagonist; GANT61 – GLI antagonist. Bars represent mean ± SEM of 5 NHK and 4 ADPKD cell lines (Summary Table S1). Cells of each line were plated in triplicate wells. Statistical significance was determined by ANOVA and Tukey’s test. *P < 0.05; ***P < 0.001; ****P < 0.0001.

Since Hh signaling affects proliferation of multiple cell types, we examined proliferation of ADPKD cells in response to Hh modulators. NHK and ADPKD cells were treated with SAG or with SMO or GLI antagonists, Sant2 or Gant61, respectively, alone or in combination with SAG, for 48 hours. Cell counts were then obtained. As control, cells of designated wells were treated with epidermal growth factor (EGF), which increases proliferation of both NHK and ADPKD cells^31^ (Fig. 5). In both NHK and ADPKD cells, SAG increased proliferation, and Gant61 and Sant2 reduced proliferation (Fig. 5). Additionally, treatment with SAG together with either Gant61 or Sant2 reduced proliferation relative to SAG (Fig. 5), suggesting specificity of the Hh modulators.

**Figure 6:**
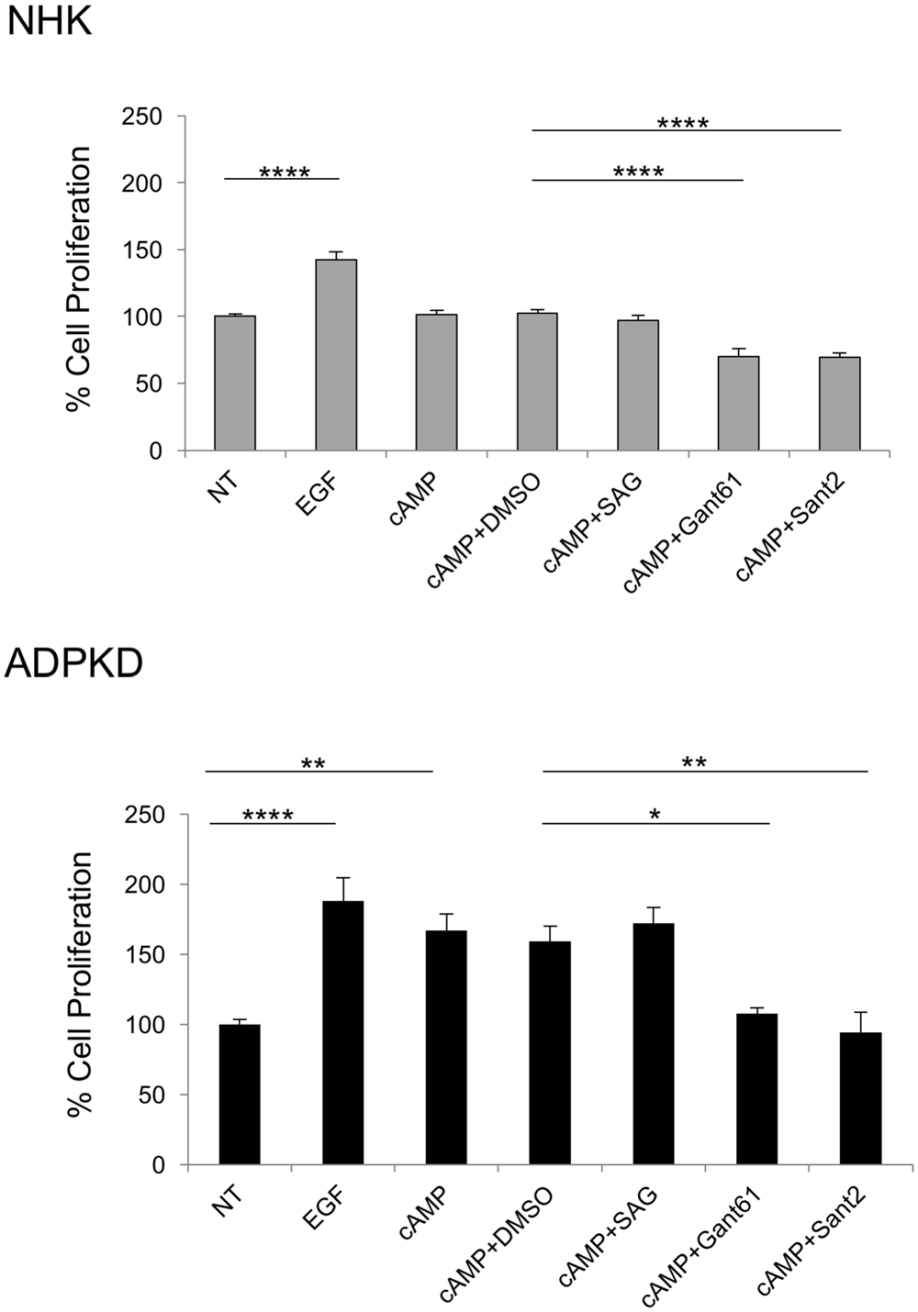
Hh inhibitors counteract proliferative effect of cAMP in human ADPKD primary renal cells. Percent cell proliferation of NHK and ADPKD cells. NT-no treatment; EGF – epidermal growth factor; SAG – Smoothened agonist; SANT2- Smoothened antagonist; GANT61 – GLI antagonist. Bars represent mean ± SEM of 3 NHK and 3 ADPKD cell lines (Summary Table S1). Cells of each line were plated in triplicate wells. Statistical significance was determined by ANOVA and Tukey’s test. *P < 0.05; **P < 0.01; ****P < 0.0001.

In ADPKD cells, but not in NHK cells, cAMP is mitogenic^32^. To determine if Hh inhibitors can mitigate cAMP-mediated proliferation, NHK and ADPKD cells were treated with cAMP in the presence of DMSO or a Hh modulator. As control in all experiments, cells of designated wells were treated with EGF or cAMP ascertaining that NHK and ADPKD cells increased proliferation in response to EGF and that ADPKD cells increased proliferation in response to cAMP (Fig. 6). In NHK cells, cAMP, cAMP with DMSO, or cAMP with SAG, did not alter cell proliferation, but treatment with cAMP together with Gant61 or Sant2 reduced proliferation (Fig. 6). In ADPKD cells, cAMP increased proliferation, and cAMP with DMSO showed similar proliferation as cAMP alone. Treatment with cAMP and SAG yielded similar cell counts as treatment with cAMP and DMSO (Fig. 6), suggesting that SAG does not increase cAMP-mediated proliferation. However, cAMP together with either Gant61 or Sant2 reduced cell counts relative to treatment with cAMP and DMSO (Fig. 6), suggesting that Gant61 or Sant2 can offset cAMP-induced proliferation. Using a Viability/Cytotoxicity Assay that incorporates calcein AM in live cells (GFP) and ethidium homodimer-1 in dead cells (RFP), we observed that treatment of NHK and ADPKD cells with Hh modulators, alone or together with cAMP, resulted in similar proportions of live and dead cells as non-treatment, indicating that the Hh modulators did not cause cell death (Supplementary Fig. S6). Together, these data demonstrate that Hh signaling can modulate proliferation of both NHK and ADPKD epithelial cells, and moreover, that Hh inhibition can counter the mitogenic effect of cAMP in ADPKD cells.

**Figure 7:**
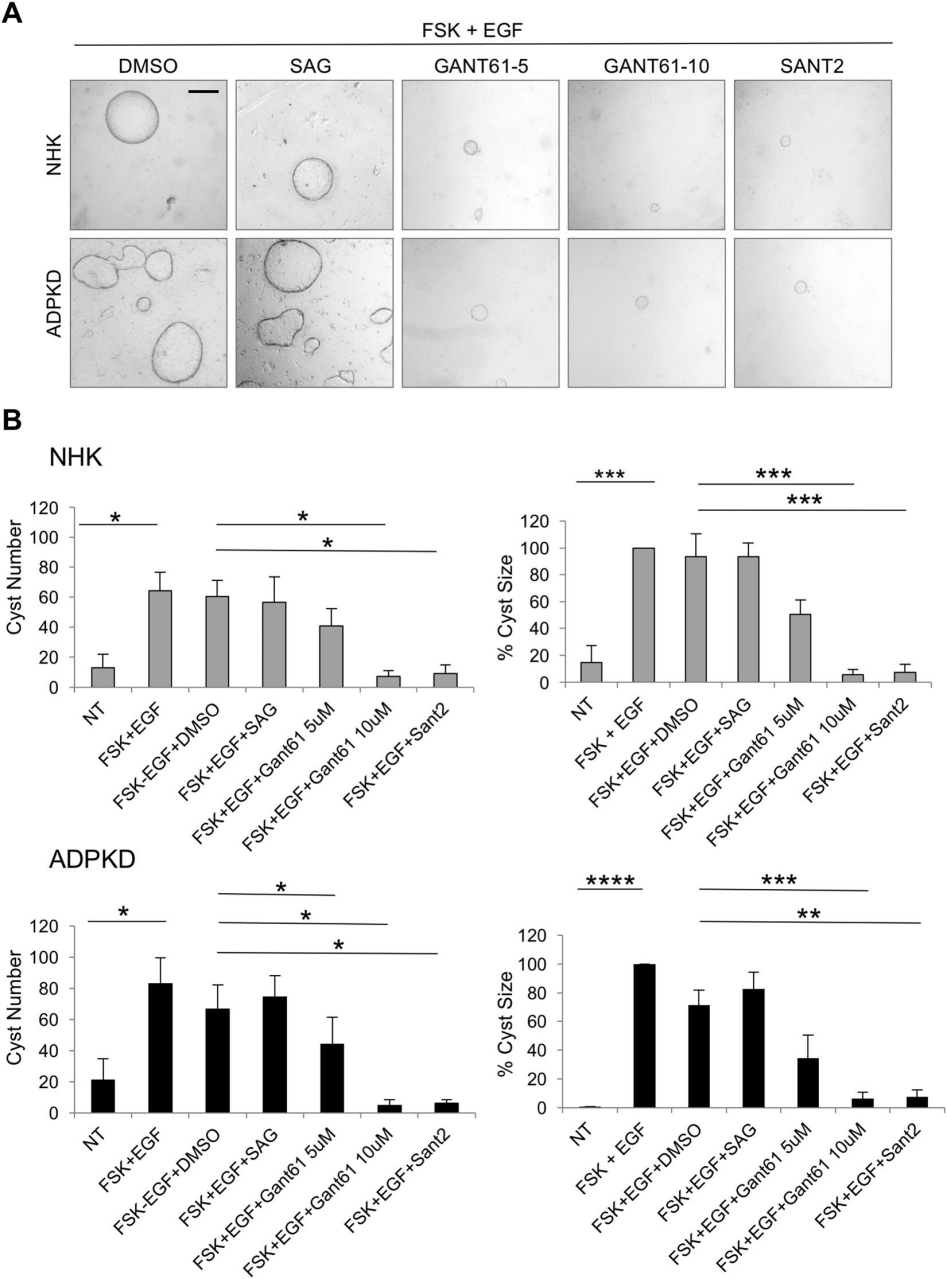
Hh inhibitors suppress microcyst formation of human primary renal epithelial cells. (**A**) Representative images of NHK and ADPKD microcysts in the presence of DMSO or Hh modulators. Cysts were imaged and quantified using Image-Pro Premier. Gant61-5 – Gant61 at 5 μM; Gant61-10 – Gant61 at 10 μM. Scale bar = 500 μm. (**B**) Quantification of microcysts. Cyst size was calculated as a proportion relative to average cyst size of FSK and EGF treatment group, which was set at 100. FSK-forskolin. Bars represent mean ± SEM of 3 NHK and 4 ADPKD cell lines (Summary Table S1). Cells of each line were plated in six replicate wells. Statistical significance was determined by ANOVA and Tukey’s test. *P < 0.05; **P < 0.01; ***P < 0.001; ****P < 0.0001.

Finally, we examined the effect of Hh modulation on microcyst formation of ADPKD and NHK cells. Microcyst formation was initiated by treatment with forskolin (FSK), which is a cAMP agonist, and EGF, which has an established role in promoting proliferation in ADPKD. Once microcysts were observed, cysts were treated with SAG, Gant61 or Sant2, alone or in combination with FSK and EGF. Continued treatment with FSK and EGF following microcyst initiation caused maximal cyst formation and growth in NHK (64.3 ± 12.37 cysts/experiment with average cyst surface area of 18.5 ± 3.93 mm^2^) and ADPKD cells (83.0 ± 16.39 cysts/experiment with average cyst surface area of 46.6 ± 21.86 mm^2^) (Fig. 7; Figure S7), while treatment with Hh modulators alone did not influence cyst formation or growth (data not shown). SAG together with FSK and EGF resulted in similar cyst number and size as FSK and EGF treatment, suggesting SAG does not exacerbate FSK and EGF-induced cyst growth (Fig. 7). Conversely, treatment with Gant61 or Sant2 together with FSK and EGF, markedly reduced number and size of microcysts relative to FSK and EGF-treated cells (Fig. 7). These data suggest that while Hh signaling is insufficient to induce cyst formation and growth, inhibition of Hh signaling within a cystic environment can mitigate cystogenic processes *in vitro*.

## Discussion

The primary cilium is important for renal tubular integrity, but the mechanisms by which ciliary dysfunction causes or modifies renal cystogenesis are not understood. Multiple mechanisms may lead to a renal cyst, and these may compare or contrast among different renal cystic diseases. ADPKD causes enlarged kidneys with varying degrees of fibrosis and ESRD in the mid-50s, while non-PKD renal cystic diseases often reduce kidney size due to abundant fibrosis and cause ESRD in the pediatric years. In ADPKD, cilia lack polycystin but are thought to be structurally intact, while in non-PKD cystic kidney disease, cilia structure is often disrupted due to mutation of a ciliary structural component. Cilia regulate Hh signaling, and this pathway is often altered in cilia mouse mutants with or without an overt ciliary structural defect^11,12,33–36^. Since others have reported increased *Gli1* in cystic kidneys of ciliary mutants, *Ift140* and *Arl13B* conditional knock-out mice^14,23^, and we have observed increased *Gli1* in cystic kidneys of *Nek8*^*jck/jck*^, *Thm1* and *Pkd1* conditional knock-out mice (on mixed strain backgrounds)^22^, suggesting a general role for elevated Hh signaling in renal cystogenesis, we investigated the Hh pathway in human ADPKD.

Previously, a transcriptome analysis of human ADPKD renal tissue revealed increased expression of Hh components, *PTCH1*, *GLI2* and *GAS1*^24^. Our data showing enhanced staining of nuclear GLI1 in interstitial and epithelial cells of ADPKD cystic tissue add to this. Renal tubular epithelial cells have been reported to express Hh ligand^18^, thus the presence of GLI1 in both interstitial and epithelial cells suggests occurrence of paracrine and autocrine Hh signaling mechanisms in ADPKD. Paracrine and autocrine Hh signaling have been reported in other kidney pathologies. Increased paracrine Hh signaling was demonstrated in renal fibrosis^18^, while elevated autocrine Hh signaling was shown in renal cell carcinoma (RCC)^16^. The intact Hh signaling we observed in NHK and ADPKD primary renal epithelial cells also supports the presence of autocrine signaling. This is consistent with studies in IMCD cells, demonstrating GLIS2 as a negative regulator of Hh signaling^20^ and showing reduced ciliary GPR161, a Hh negative modulator, in response to SAG^37^, collectively indicating that the Hh signaling machinery is present in kidney epithelial cells. We anticipated increased Hh signaling in ADPKD primary renal epithelial cells similar to ADPKD renal tissue. This was not the case, which could reflect that additional cell types or signaling molecules cross-talk *in vivo* to cause upregulation of GLI1. Indeed, a caveat of *in vitro* systems is the inability to mimic all conditions present *in vivo*. Yet, *in vitro* models can be useful in understanding how cells might respond to a particular stimulus, and the primary renal epithelial cells allow functional analyses on patient-derived materials, which cannot be done at the tissue level.

Our data show that Hh inhibitors can abate cAMP-mediated proliferation in ADPKD cells and FSK and EGF-induced cystogenesis *in vitro*. Previously, we observed that Gant61 and Sant2 reduced the cAMP-mediated cystogenic effects in cultured mouse embryonic kidneys^22^. Collectively these data suggest that Hh signaling is a necessary component of cAMP-mediated proliferation and cystogenesis in these systems. In human ADPKD cells, elevated intracellular cAMP activates cystic fibrosis transmembrane conductance regulator (CFTR)-mediated Cl^−^ secretion and B-Raf/MEK/ERK signaling, causing fluid secretion and increased proliferation of cyst-lining epithelial cells, respectively^38,39^. ADPKD cells have low homeostatic intracellular Ca^2+^, which causes the mitogenic effect of cAMP, while augmenting intracellular Ca^2+^ in ADPKD cells counters this effect^40^. Hh inhibition may mitigate the proliferative and cystogenic effects of cAMP, FSK and EGF, by possible crosstalk between Hh and ERK signaling, which has been observed in RCC and other cancers^16,41,42^. Alternatively, Hh inhibition by a SMO inhibitor, GCD-0449, has been shown to increase steady-state levels of intracellular Ca^2+^ in a lung cancer cell line^43^. Similarly, Hh inhibition might also increase intracellular Ca^2+^ in kidney epithelial cells.

Alternatively, Hh signaling and polycystin function might intersect at the primary cilium. PC2 has been indicated to be part of a complex in the cilium with adenylyl cyclases 5 and 6, and phosphodiesterase 4 C, which synthesize and catabolizes cAMP^44^, respectively, likely regulating cAMP at the primary cilium. Advanced imaging technology has enabled visualization of ciliary pools of Ca^2+^ and cAMP^45,46^. In mouse embryonic fibroblasts, Hh activation increased ciliary Ca^2+ ^^45^, and decreased ciliary cAMP^46^. These studies also demonstrated that ciliary and intracellular pools of Ca^2+^ and cAMP are distinct. Since Hh inhibition by GDC-0449 increased intracellular Ca^2+^ in a lung cancer cell line^43^, Hh activity may have opposing effects on ciliary and intracellular Ca^2+^. Thus, regulation of Hh signaling on ciliary and intracellular Ca^2+^ requires further investigation.

SAG alone, but not SAG with cAMP, increased proliferation of NHK cells. This may suggest that cAMP countered the effect of SAG and inhibited Hh signaling in these cells. Yet treatment with the Hh inhibitors together with cAMP reduced cell proliferation relative to cAMP with DMSO, suggesting that proliferation of NHK cells may require a certain level of Hh signaling, which was inhibited by Gant61 and Sant2. In mouse PKD kidneys, which have high levels of cAMP, *GLI1* is increased indicating enhanced Hh signaling^22^. Thus, while cAMP might dampen the Hh pathway in certain contexts, this dampening effect may be overridden in the PKD setting.

Two studies have documented increased ciliary length in *Pkd* models^25,26^, which prompted us to examine IFT and BBS localization in ADPKD. Normal localization of IFT and BBSome components in several ADPKD cell lines indicates that the ciliary trafficking machinery is not affected by polycystin dysfunction. Thus other mechanisms may account for the increased ciliary length reported in these *Pkd* models. Polymorphisms in the mouse strain background may interact with the *Pkd* mutation to increase cilia length. These polymorphisms might reside in genes that regulate ciliary structure^4^, cAMP^47^, cytosolic tubulin, actin machinery^37^, or other factor that can modulate cilia length. These genes would represent PKD modifiers.

Aside from IFT, epigenetic regulation can also result in increased GLI levels. Epigenetics has been shown to affect PKD in a preclinical model, and cancer studies have linked epigenetics to Hh regulation. Inhibition of Brd4, a BET bromodomain protein and epigenetic regulator, attenuated PKD progression in a *Pkd1* conditional knock out mouse^48^. In addition, treatment of a medulloblastoma mouse model with I-BET151, a Brd4 inhibitor, reduced *Gli1* expression, cell proliferation and tumor growth, indicating epigenetics drives Hh activation in cancer^49^. *GLI1* may be a target of BRD4 in ADPKD as well.

Since some, but not all, cyst-lining epithelial cells in ADPKD renal tissue showed increased nuclear GLI1 staining, and increased *Gli1* transcripts have been evident in kidneys of mouse models that were already cystic^14,22,23^, we speculate that Hh signaling may increase with disease progression, and that patients with more advanced ADPKD might show a greater increase in Hh signaling than those with earlier disease. Importantly, Hh inhibitors countered the proliferative and cystogenic effects of cAMP/forskolin in patient-derived renal epithelial cells. Our findings suggest clinical relevance of the Hh pathway in ADPKD. An important experiment will be to examine whether Hh inhibition attenuates disease in an appropriate *Pkd* model *in vivo*. Hh inhibition may also serve to attenuate fibrosis^18^, which is a significant component of ADPKD pathology. Still, multiple pathways are misregulated in ADPKD, and crosstalk and feedback loops may also be present. Thus targeting more than one pathway at different disease stages might be most effective. Interestingly, Hh and ERK signaling have been demonstrated to work cooperatively in some cancers, with inhibition of one pathway showing modest effect, but simultaneous inhibition of both pathways having a synergistic effect^42,50–54^. Exploring similar possibilities in appropriate *Pkd* models *in vivo* may help determine effective combinatorial therapeutic strategies against ADPKD.

## Methods

### ADPKD tissue and primary cells

Human ADPKD and NHK tissues and primary cells were obtained from the PKD Biomarkers and Biomaterials Core at the University of Kansas Medical Center (KUMC)^55^. ADPKD kidneys were obtained from the KU hospital and hospitals participating in the Polycystic Kidney Research Retrieval Program with the assistance of the PKD Foundation (Kansas City, MO) and the Biospecimen Shared Resource (BSR) at KUMC. These kidneys were removed solely for clinical purposes and de-identified prior to being submitted to the Repository; therefore, the use of the materials is not considered human subjects research. The protocol for the use of ADPKD tissue for research was approved by the Institutional Review Board at KUMC. These patients were below the age of 60 years. Since the majority of the ADPKD cases is caused by mutation in *PKD1* and has an earlier onset of ESRD compared to patients with *PKD2* mutation (54 vs 74 years)^56^, all of the primary ADPKD cells were likely derived from *PKD1*-mutant kidneys. Normal regions of human kidneys, confirmed by histological examination, were obtained from nephrectomy specimens through the BSR at KUMC. Normal kidneys withheld from transplantation due to poor perfusion characteristics and anatomical abnormalities were obtained from the Midwest Transplant Network (Kansas City, KS).

The protocol used to generate NHK and ADPKD primary renal epithelial cells has been detailed previously^27,28,31,57–59^. Samples are retrieved from the renal cortex of NHK individuals and from the epithelium of surface (cortical) cysts of ADPKD individuals. Cells from several cysts are pooled together. Preparative steps, such as collagenase treatment and keeping cell passage numbers ≤2, which collectively keep lines fibroblast-free, are identical between NHK and ADPKD cells. The majority of ADPKD and NHK cells have been shown to stain positively for *Dolichos biflorus* agglutinin, suggesting that most cells derive from collecting ducts^27,28^. The Core provides approximately 1–2 million primary cells each of 1 NHK and 1 ADPKD line on a weekly basis to investigators.

### qPCR

RNA was extracted using Trizol (Life Technologies) and RNA integrity was verified by the Genome Sequencing Facility at the KUMC. RIN values ranged from 8–10. One microgram of RNA was converted into cDNA using Quanta Biosciences qScript cDNA mix (VWR International). The analysis was made using Quanta Biosciences Perfecta qPCR Supermix (VWR International) and a BioRad CFX Connect Real-Time PCR Detection System. Primers used were *GLI1* (Forward: 5′ CAG GGA GGA AAG CAG ACT GA 3′; Reverse: 5′ ACT GCT GCA GGA TGA CTG G 3′), *GLI2* (Forward: 5′ CAC GCT CTC CAT GAT CTC TG 3′; Reverse: CCC CTC TCC TTA AGG TGC TC 3′), *GLI3* (Forward: 5′ CGA ACA GAT GTG AGC GAG AA 3′; Reverse: 5′ GTC TGT CCA GGA CTT TCA TCC T 3′) and housekeeping gene *OAZ1* (Forward: 5′ CAC CAT GCC GCT CCT AAG 3′; Reverse: GAG GGA GAC CCT GGA ACT CT 3′). qPCR was performed on RNA lysates of three ADPKD and three NHK cell lines.

### Western blot

Protein extracts were obtained by homogenizing frozen kidney tissue with Passive Lysis Buffer (Promega) cointaining proteinase inhibitor cocktail (Pierce) using Bullet Blender Bead Lysis tubes (MidSci) and a Bullet Blender Storm 24 (Next Advance) set at Speed 10 for approximately 10 minutes, centrifuging lysates at 4 °C at maximum speed for 1 minute, and collecting the supernatant. Western blot was done as described^22^, using primary antibodies for GLI1 and βactin (Cell Signaling Technology). Extracts from six NHK and five ADPKD frozen renal tissue samples were examined for GLI levels.

### Immunohistochemistry

Human ADPKD and NHK renal tissue sections were deparaffinized in xylene and rehydrated through an ethanol series to distilled water. Antigen retrieval was performed by steaming tissue sections for 25 minutes in Sodium Citrate Buffer (10 mM Sodium Citrate (Fisher Scientific), 0.05% Tween 20 (Fisher Scientific) in autoclaved water, pH 6.0). To minimize background staining, sections were treated with 3% hydrogen peroxide for 30 min, washed in PBS, then blocked with 1% BSA for 1 hour. Cells were then incubated with GLI1 antibody (Cell Signaling) overnight at 4 °C. Following 3 washes in PBS, sections were incubated with HRP-conjugated rabbit secondary antibody (Cell Signaling) for 30 minutes. Following another 3 washes in PBS, tissues were incubated with ABC reagent (Vector Laboratories), rinsed in PBS, and then incubated with SigmaFAST DAB metal enhancer (Sigma) until desired signal/color was obtained. To determine GLI1 localization in proximal tubules or collecting ducts, sections were incubated with fluorescein-conjugated *Lotus tetragonolobulus* or *Dolichus biflorus* agglutinin for 1 hour at room temperature, washed and mounted in Vectashield containing 4,6-diamidino-2-phenylindole (DAPI) (Vector Laboratories). Staining was visualized and imaged using a Nikon 80i light/fluorescent microscope and Nikon DS-Fi1 camera. Immunohistochemistry images were converted to grayscale.

### Immunofluorescence

Cells were washed with PBS, fixed with 4% paraformaldehyde and 0.2% triton X-100 for 10 minutes at room temperature, washed with PBS, and blocked with 1% BSA in PBS for 1 hour, and then incubated with antibodies against SMO (generous gift from Dr. K Anderson, and purchased from Abcam), IFT52, IFT81, IFT88, IFT140, BBS2, BBS5 (Proteintech) and acetylated α-tubulin (Sigma) overnight at 4 °C. To address localization of the SMO within cilia, cells were first treated with 500 nM SAG (Enzo Life Sciences) in DMSO. Following 3 washes in PBS, cells were incubated with anti-rabbit AF488 and anti-mouse AF594 (InVitrogen Technologies) for 30 minutes at room temperature. Cells were washed 3X in PBS, and mounted with Vectashield containing 4,6-diamidino-2-phenylindole (DAPI) (Vector Laboratories). Immuno-labeled cells were viewed and imaged using a Leica TCS SPE confocal microscope configured on a DM550 Q upright microscope. Each ciliary antibody was examined in a minimum of three NHK and three ADPKD cell lines.

### Cell proliferation

ADPKD or NHK cells (15,000 cells/well) were plated in a 24-well plate (Costar) in DMEM/F12 media containing 1% FBS, ITS (Insulin, Transferrin, Selenium) culture supplement (Fisher) and Pen/Strep and grown to approximately 70% confluency. Cells were serum-starved in DMEM/F12 media containing 0.02% FBS and Pen/Strep overnight. The following morning, cells were treated with 500 nM SAG, 5 μM Gant 61 or Sant2 (Enzo Life Sciences), 100 μM cAMP (Sigma), 100ng/mL EGF (Sigma), DMSO (Sigma) alone or in combination for 48 hours. Cells were trypsinized in 100 μl of Trypsin (Gibco), and 10 μl were counted in a cell counting slide (Bio-Rad) using a TC20 automated cell counter (Bio-Rad). Assays were performed in triplicate wells, using primary cells from at least three NHK and three ADPKD kidneys.

### Viability/Cytotoxicity Assay

Following the same 48-hour treatment of NHK and ADPKD cells with Hh modulators, alone or with cAMP, as done in the cell proliferation assays, the LIVE/DEAD Viability/Cytotoxicity Kit for mammalian cells (InVitrogen) was used according to manufacturer’s instructions. Cells were imaged using a digital camera attached to an inverted microscope Nikon Eclipse TE2000-U. Assays were performed in duplicate wells, using primary cells from three NHK and three ADPKD kidneys.

### Microcyst Assay

ADPKD and NHK cells (4,000 cells/well) were dispersed in cold Type I collagen (Advanced Biomatrix; San Diego, CA) in wells of a 96-well plate, warmed to 37 °C to enable polymerization of the collagen gel. Defined media (DMEM/F12, Pen/Strep, ITS Culture Supplement (Fisher), 5 × 10^−8^ M Hydrocortisone and 5 × 10^−12^ M Triiodothyronine) supplemented with forskolin (5 μM) and EGF (5 ng/ml), to stimulate *in vitro* cyst formation, was placed onto collagen-suspended cells, and refreshed every 2-3 days over a 7-day period. Once microscopic cysts (microcysts) were observed, media was replaced with media containing either forskolin, EGF, DMSO (Sigma), 500 nM SAG, 5μM or 10μM Gant 61 or 5μM Sant2 (Enzo Life Sciences) in combination or individually. For wells treated with Hh modulators alone, wells were incubated with defined media for 4 h prior to treatment with Hh modulators, to wash out initial EGF and forskolin from the collagen matrix. Following microcyst assay, culture gels were fixed in 0.5% paraformaldehyde, and microcysts were photographed with a digital camera attached to an inverted microscope Nikon Eclipse TE2000-U, objective (2×). Diameters of spherical cysts with distinct lumens were measured using Image-Pro Premier 9.2 64 bit. Assays were performed in six replicate wells, using primary cells from three NHK and three ADPKD kidneys.

### Data availability statement

Datasets generated during the current study are available from the corresponding author on reasonable request.

## Electronic supplementary material

Supplementary Info
