# Supplementary material for: Inhibition of Hedgehog signaling suppresses proliferation and microcyst formation of human Autosomal Dominant Polycystic Kidney Disease cells
Source: Sci Rep. 2018 Mar 21;8:4985. doi: 10.1038/s41598-018-23341-2 (PMC5862907; doi:10.1038/s41598-018-23341-2)
Supplement: Supplementary file 1 — Supplementary Info [file 41598_2018_23341_MOESM1_ESM.pdf]

Inhibition of Hedgehog signaling suppresses proliferation and microcyst formation of human Autosomal Dominant Polycystic Kidney Disease cells

Luciane M Silva[1, 5], Damon T Jacobs[1, 5], Bailey A Allard[1, 5], Timothy A Fields[2, 5], Madhulika Sharma[4, 5], Darren P Wallace[3,4,5] and Pamela V Tran[1,5]

[1]Dept. of Anatomy and Cell Biology, University of Kansas Medical Center, Kansas City, KS
[2]Dept. of Pathology and Laboratory Medicine, University of Kansas Medical Center, Kansas City, KS
[3]Dept. of Molecular and Integrative Physiology, University of Kansas Medical Center, Kansas City, KS
[4]Dept. of Internal Medicine, University of Kansas Medical Center, Kansas City, KS
[5]Kidney Institute, University of Kansas Medical Center, Kansas City, KS

Supplementary Tables S1 and Figures S1-S7

		NHK	ADPKD
Figure 1	Tissue 1	K235	K389
	Tissue 2	K241	K294
	Tissue 3	K395	K397
	Tissue 4	K364	K288
	Tissue 5	K378	K399
	Tissue 6	K388	
Figure 3	IFT52	K340	K315
		K388	K276
		K376	K354
	IFT81	K347	K428
		K362	K276
		K376	K315
	IFT88	K376	K276
		K265	K251
		K337	K315
	IFT140	K340	K276
		K376	K315
		K419	K417
	BBS2 and BBS5	K340	K276
		K388	K354
		K419	K417
	BBS5	K376	K315
Figure 4	qPCR	K337	K315
		K340	K354
		K388	K426
	SMO	K376	K339
		K337	K288
		K340	K276
		K347	K428
		K419	K417
Figure 5	proliferation	K291	K276
		K337	K319
		K340	K339
		K342	K346
		K419	
Figure 6	cAMP proliferation	K250	K276
		K340	K319
		K342	K339
Figure 7	microcyst assay	K340	K276
		K342	K348
		K362	K350
			K354

Table S1. Sample numbers used in experiments.

		NHK	ADPKD
Figure S5	DBA/LTL/THP	K340	K441
		K419	K386
		K325	K315
		K337	K339
		K343	K417
Figure S6	Live/Dead Assay	K340	K348
		K337	K339
		K388	K386

Table S1. Sample numbers used in experiments.

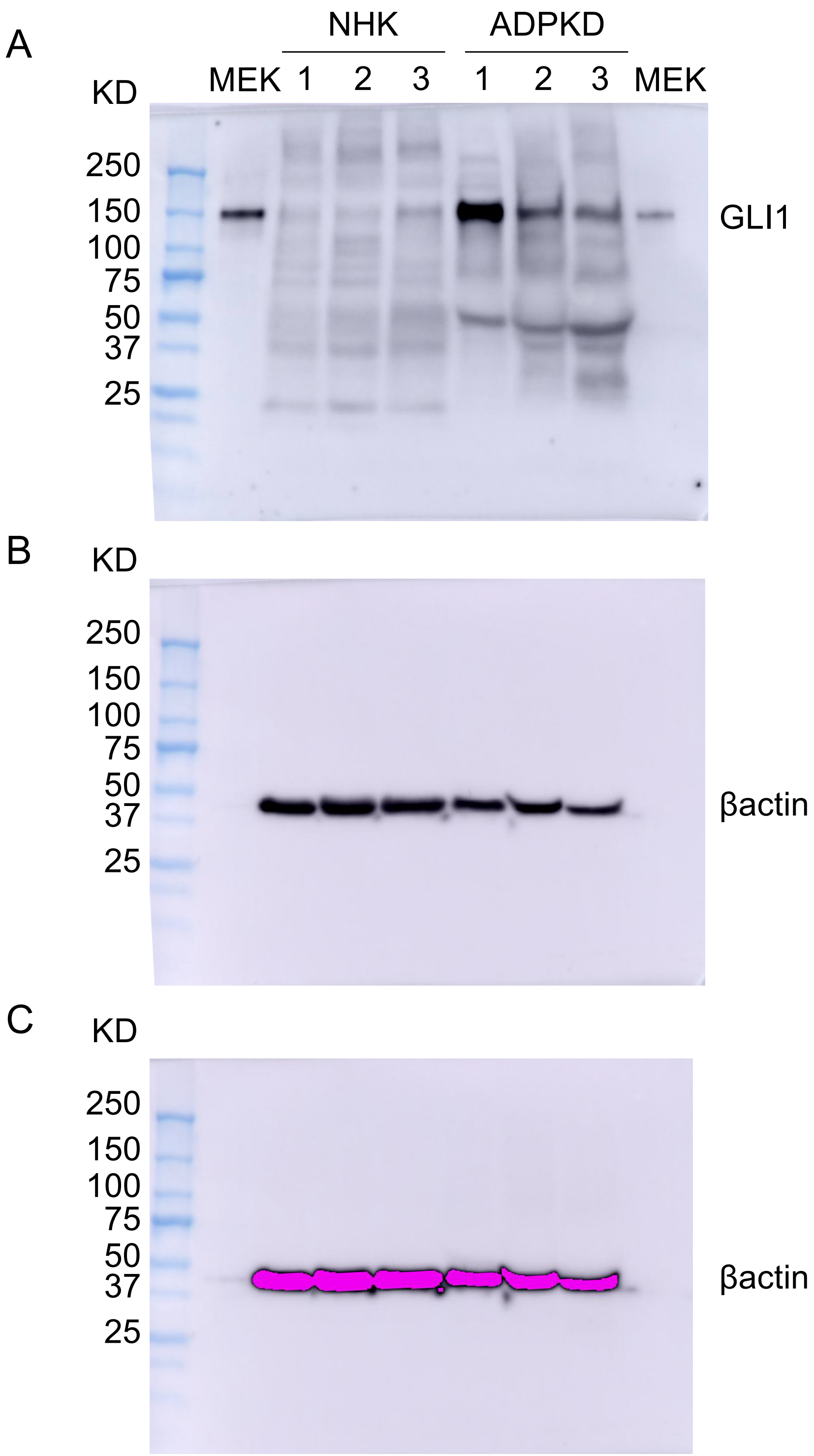

Figure S1. GLI1 is upregulated in human ADPKD renal tissue. Full-length Western blots of Fig 1. Extracts of mouse embryonic kidney (MEK) were included as a positive control for GLI1 detection. Twice as much MEK extract was loaded in lane 1 than in lane 8. A) 1-minute exposure for GLI1. B) 0.5-second exposure for ß-actin. C) 40-second exposure for ß-actin. Longer exposure of ß-actin reveals presence of band in MEK extract lane 1. This low ß-actin expression indicates markedly higher GLI1 expression in mouse embryonic kidney than in human adult kidney. Pink bands indicate over-exposure.

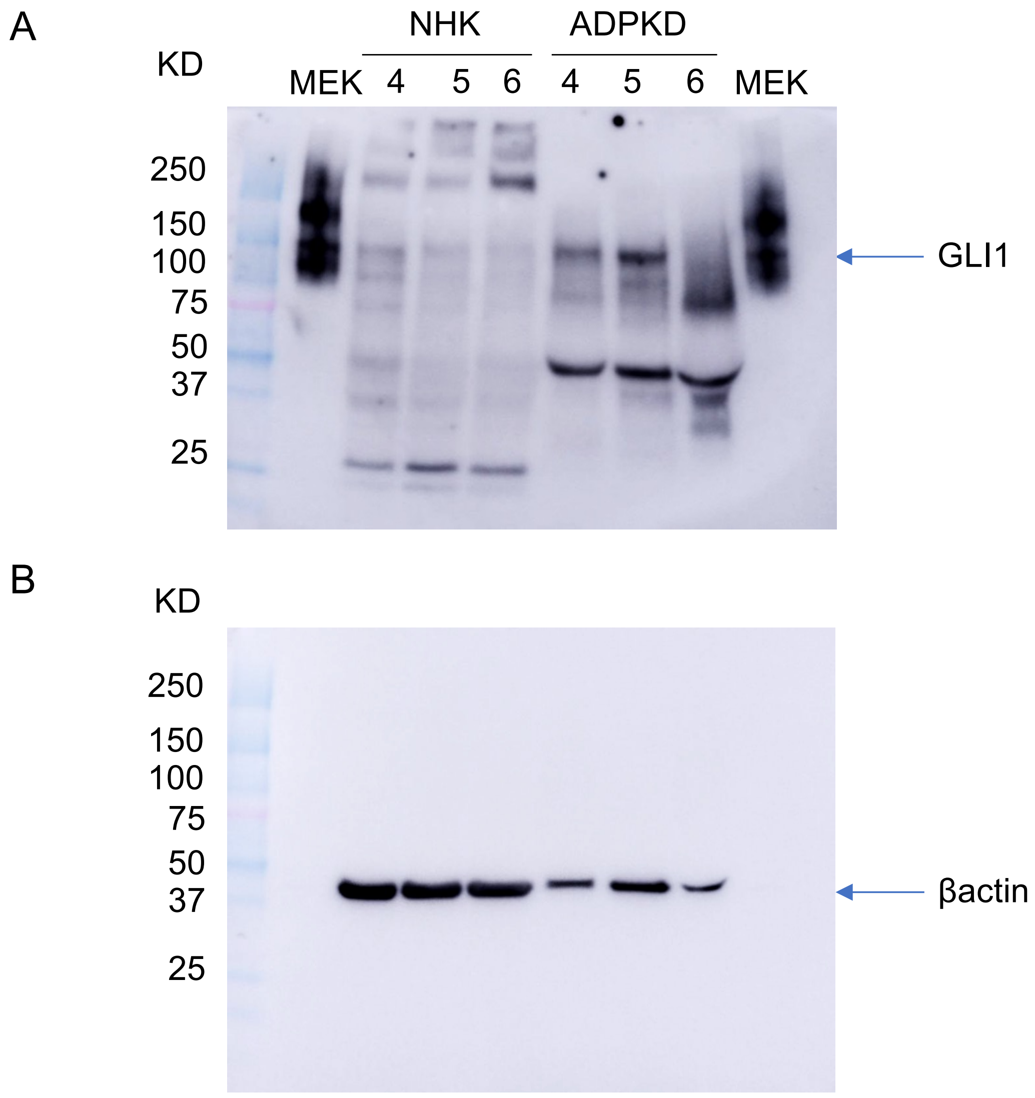

Figure S2. GLI1 is upregulated in human ADPKD renal tissue. Full-length Western blots of extracts of NHK and ADPKD renal tissue, and mouse embryonic kidney (MEK), included as a positive control for GLI1 detection. A) 1-minute exposure for GLI1. ADPKD sample 6 appears degraded, and therefore, was not included in the GLI1 quantification in Fig 1A. B) 0.5 second exposure for ß-actin. Absence of ß-actin bands in MEK lanes indicates markedly higher GLI1 expression in mouse embryonic kidney than in human adult kidney.

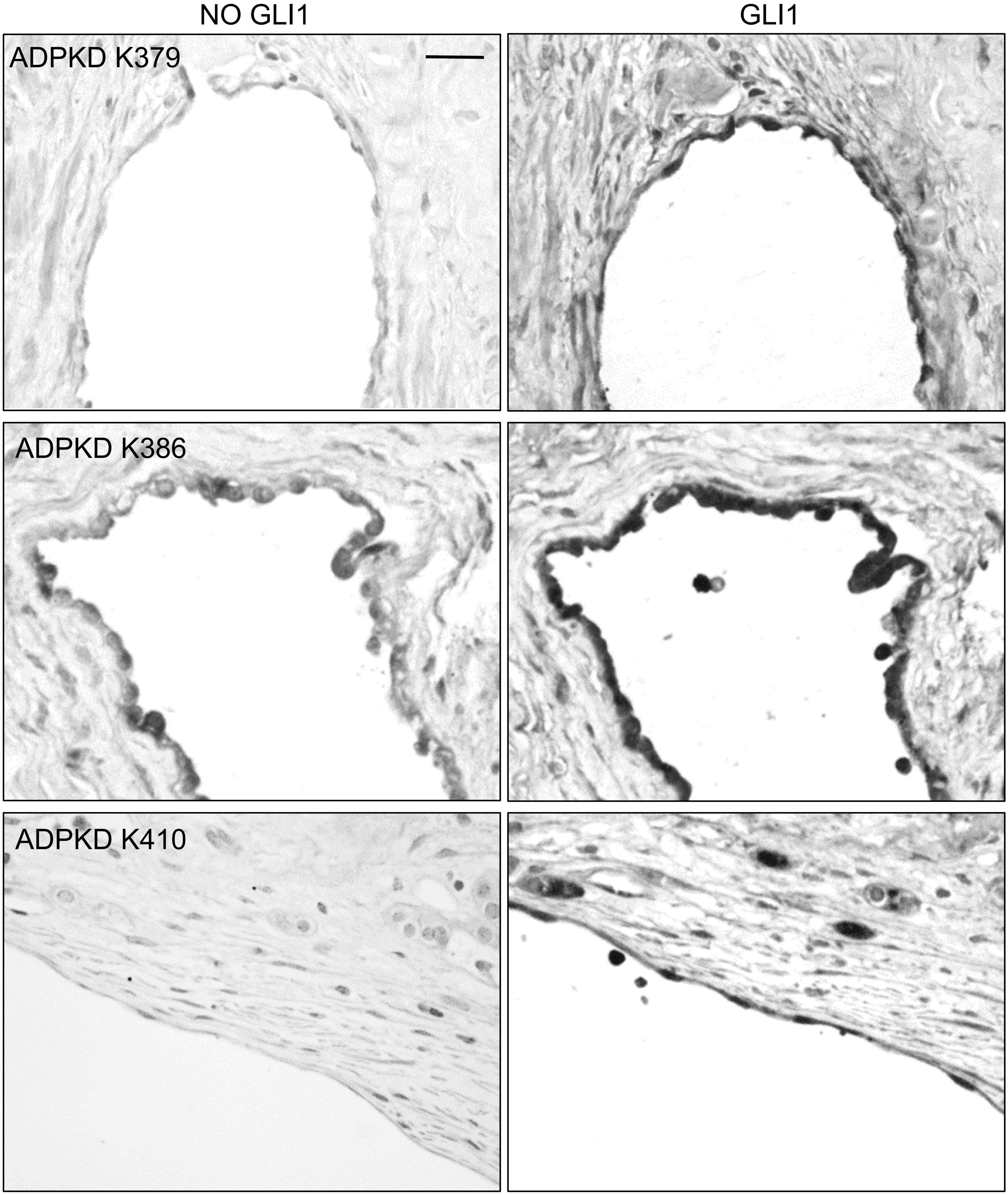

Figure S3. GLI1 immunohistochemistry on ADPKD tissue. GLI1 immunohistochemistry with no GLI1 antibody control. Images obtained with a 40X objective lens. Scale bar = 50 μm.

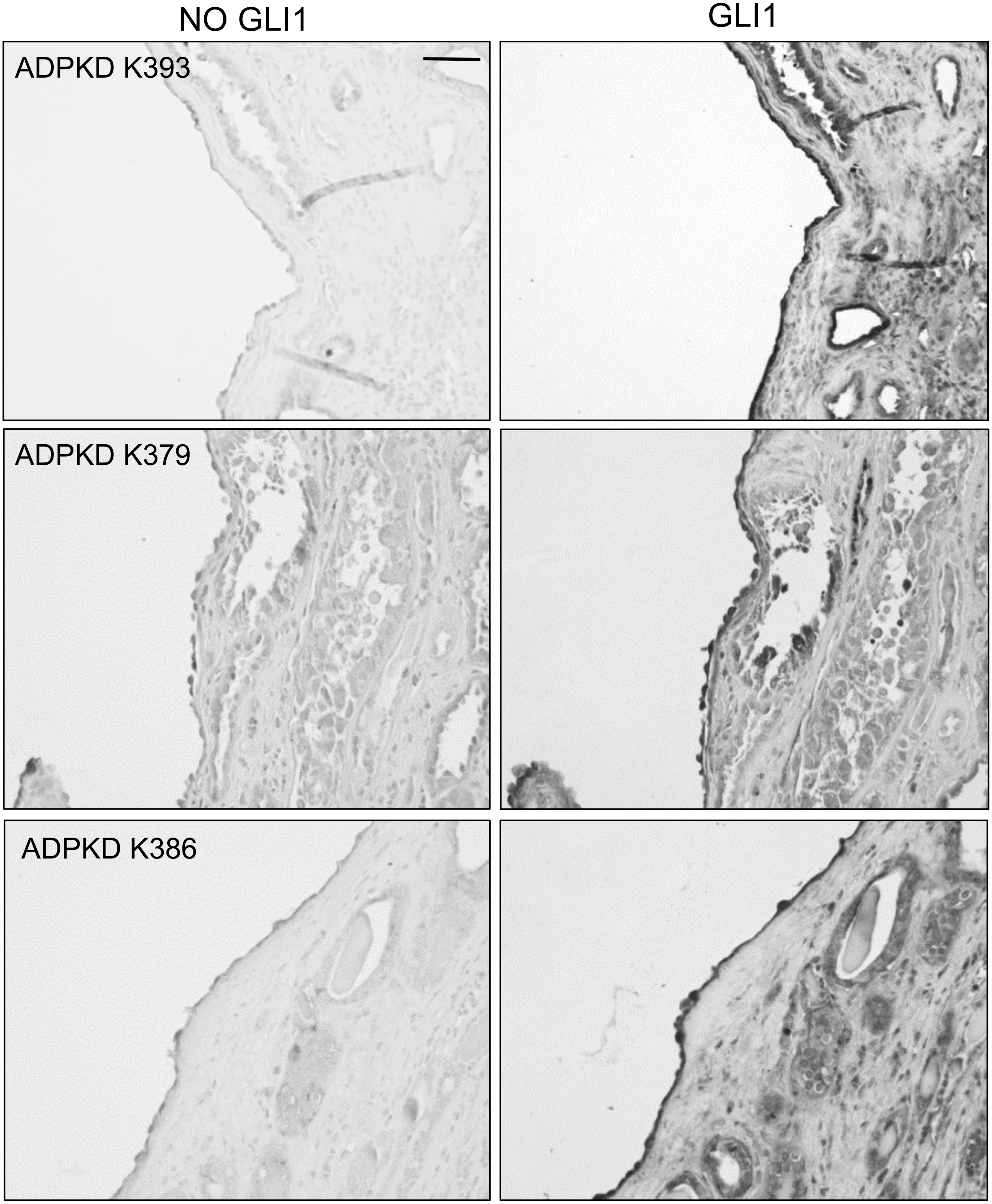

Figure S4. GLI1 immunohistochemistry on ADPKD tissue. GLI1 immunohistochemistry with no GLI1 antibody control. Images obtained with a 20X objective lens. Scale bar = 100μm.

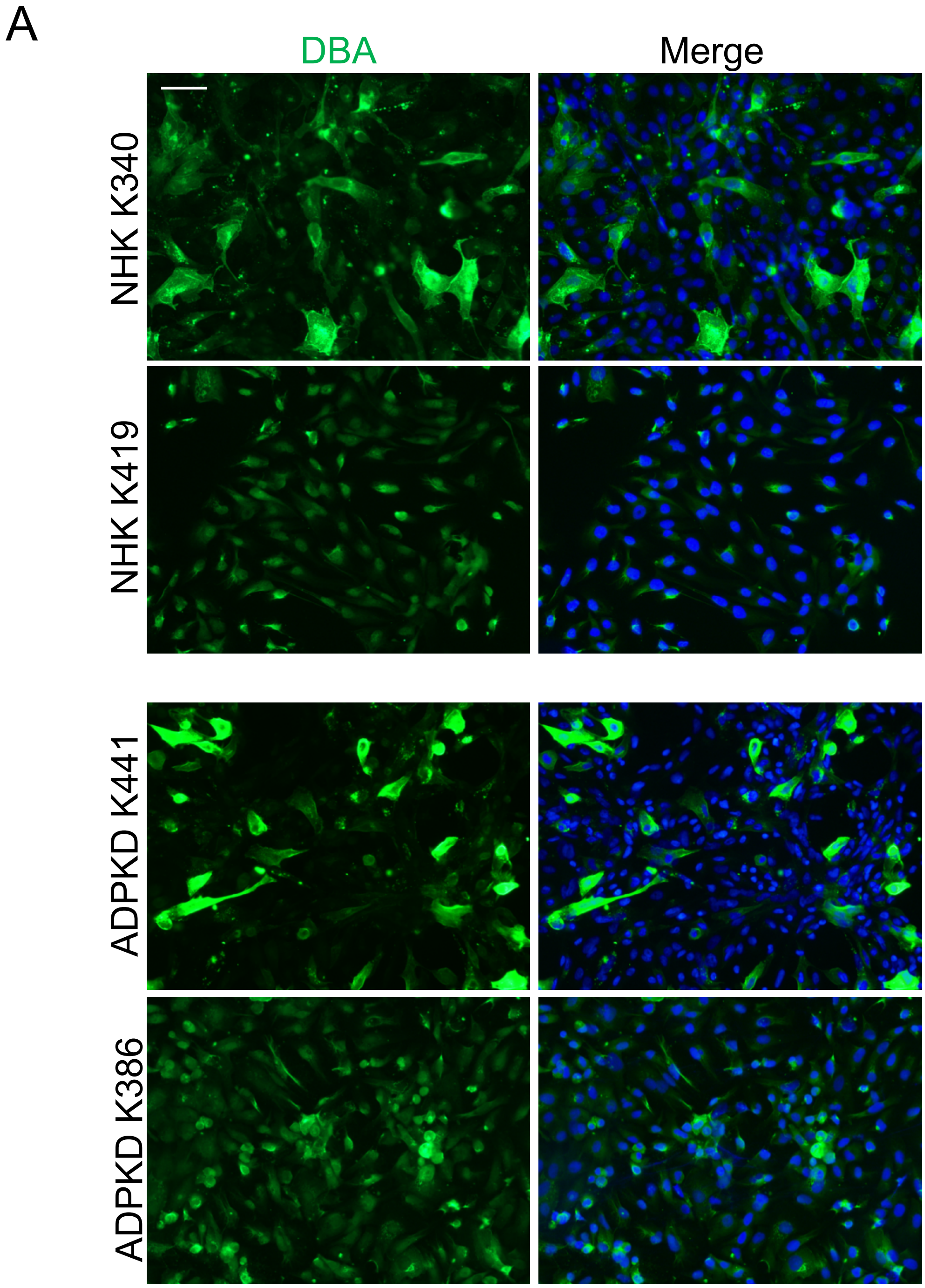

Figure S5. NHK and ADPKD primary renal epithelial cells are DBA-positive. Representative staining of primary renal epithelial cells with A) DBA or B) LTL together with immunostaining for THP, which are markers of collecting duct, proximal tubule, and Loop of Henle, respectively. Staining was performed in 5 NHK and 5 ADPKD cell lines (Summary Table S1). Scale bar = 100μm.

B

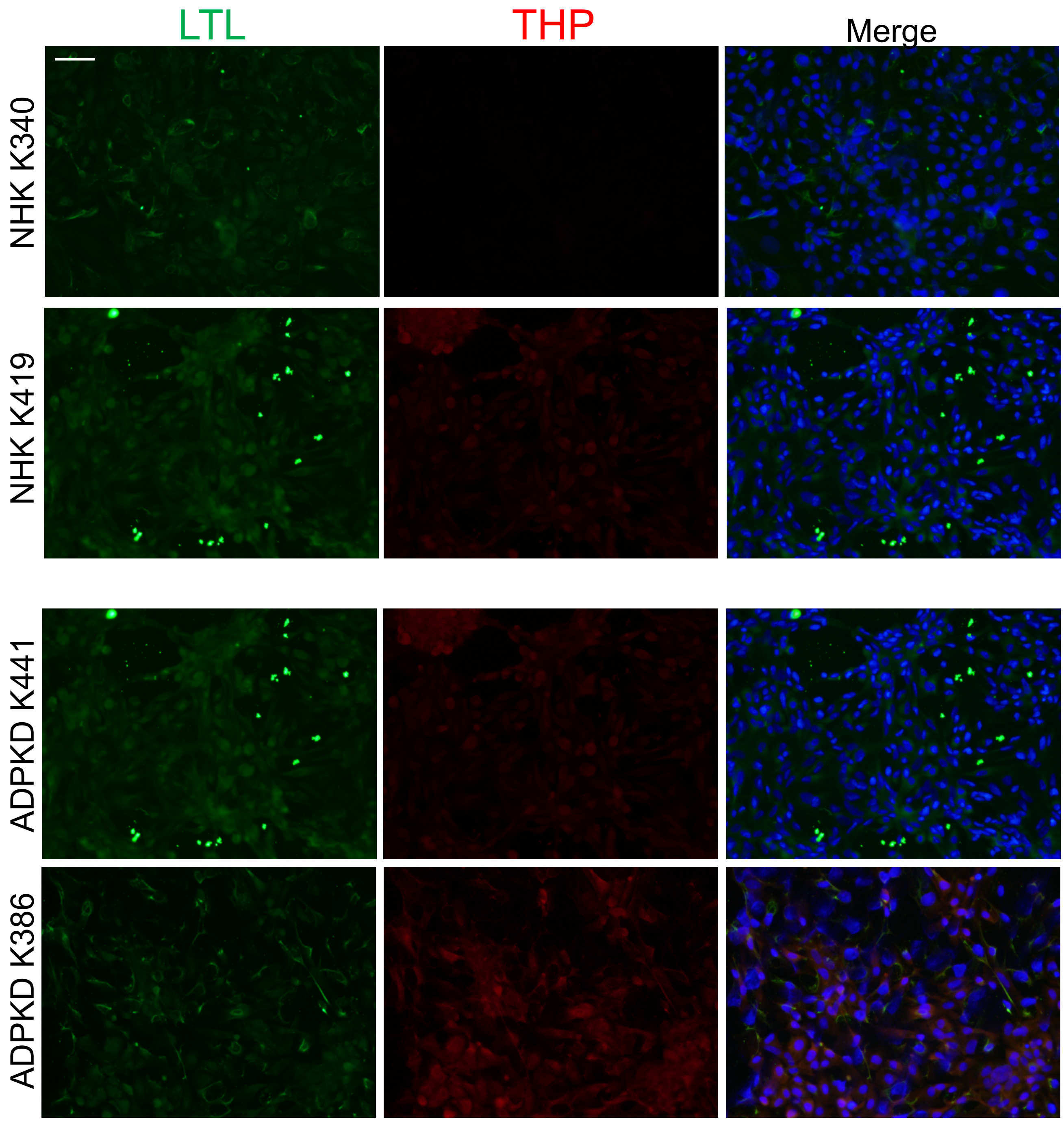

Figure S5. NHK and ADPKD primary renal epithelial cells are DBA-positive. Representative staining of primary renal epithelial cells with A) DBA or B) LTL together with immunostaining for THP, which are markers of collecting duct, proximal tubule, and Loop of Henle, respectively. Staining was performed in 5 NHK and 5 ADPKD cell lines (Summary Table S1). Scale bar = 100µm.

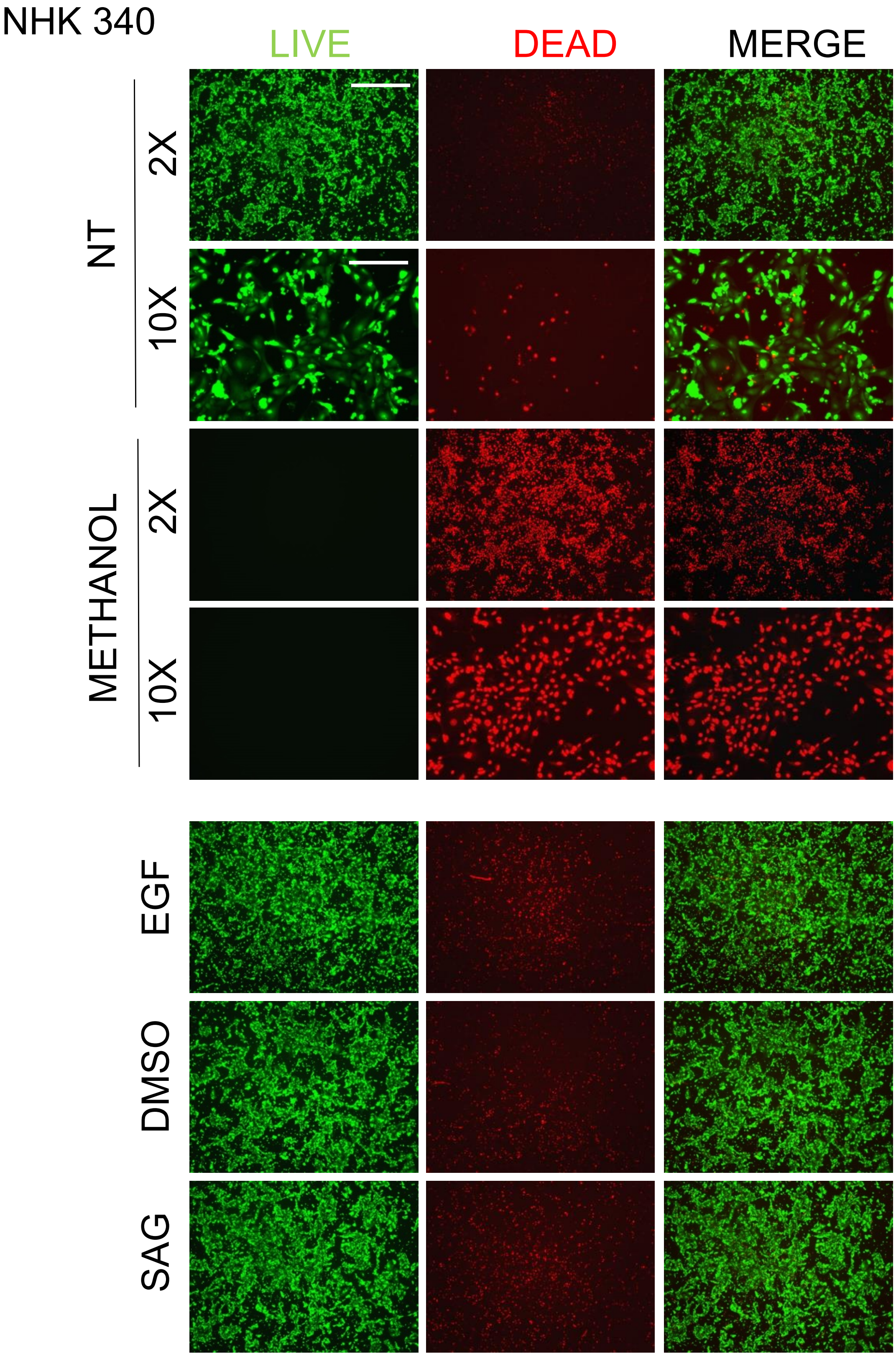

Figure S6. Analysis of cell viability and cytotoxicity of Hh modulators. NHK and ADPKD cells were treated with Hh modulators, alone or together with cAMP, then analyzed for viability/ retention of calcein AM (GFP) in membranes or for non-viability/incorporation of ethidium homodimer-1 (RFP) in nuclei. NT - no treatment. Methanol – 30-minute incubation in 70% methanol was used to cause 100% cell death. Hh treatments were imaged using a 2X objective lens. Scale bar for 2X – 500µm. Scale bar for 10X - 100µm. Assays were performed in 3 NHK and 3 ADPKD cell lines (Summary Table S1).

NHK 340

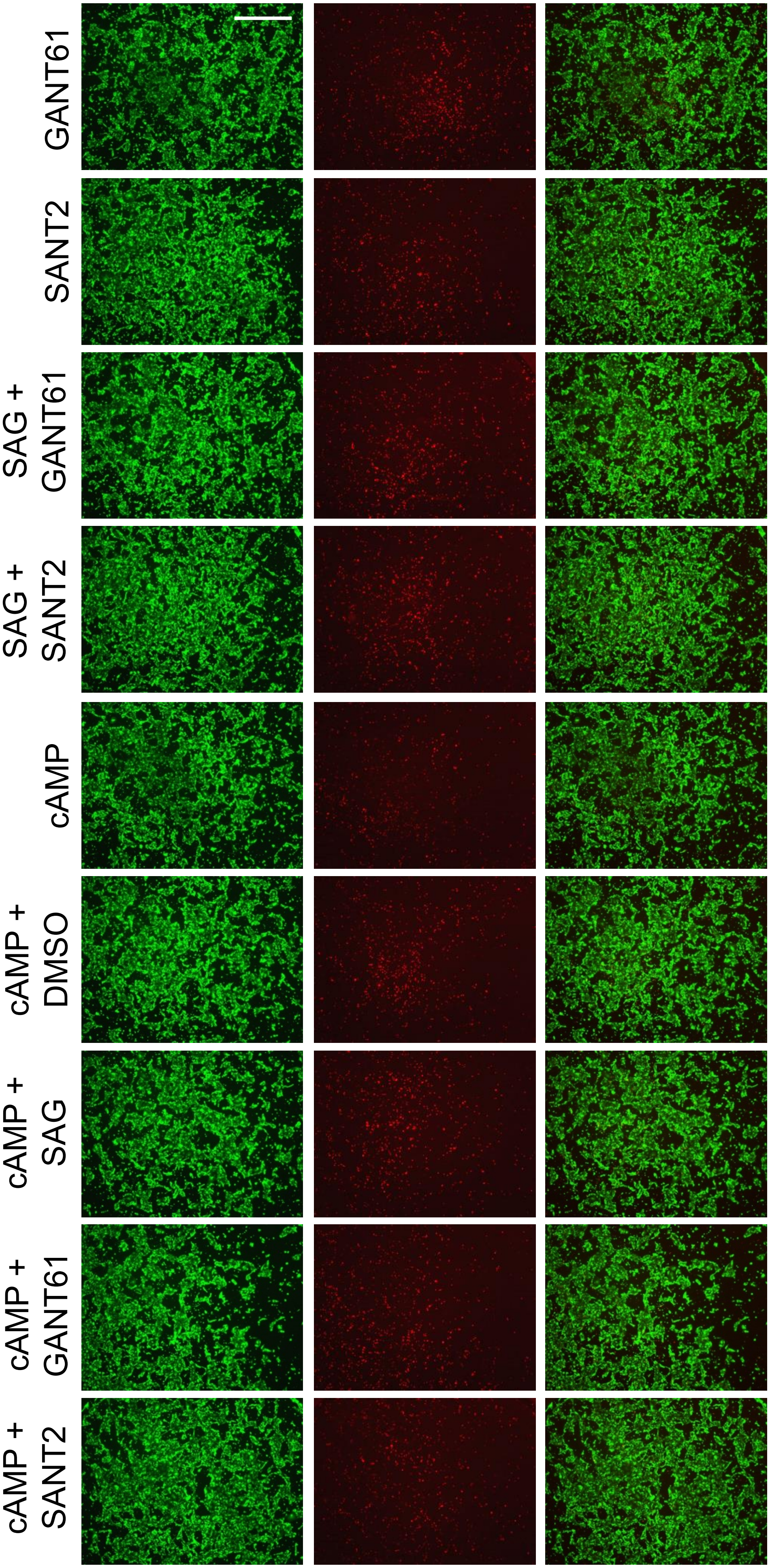

GANT61
SANT2
SAG + GANT61
SAG + SANT2
cAMP
cAMP + DMSO
cAMP + SAG
cAMP + GANT61
cAMP + SANT2

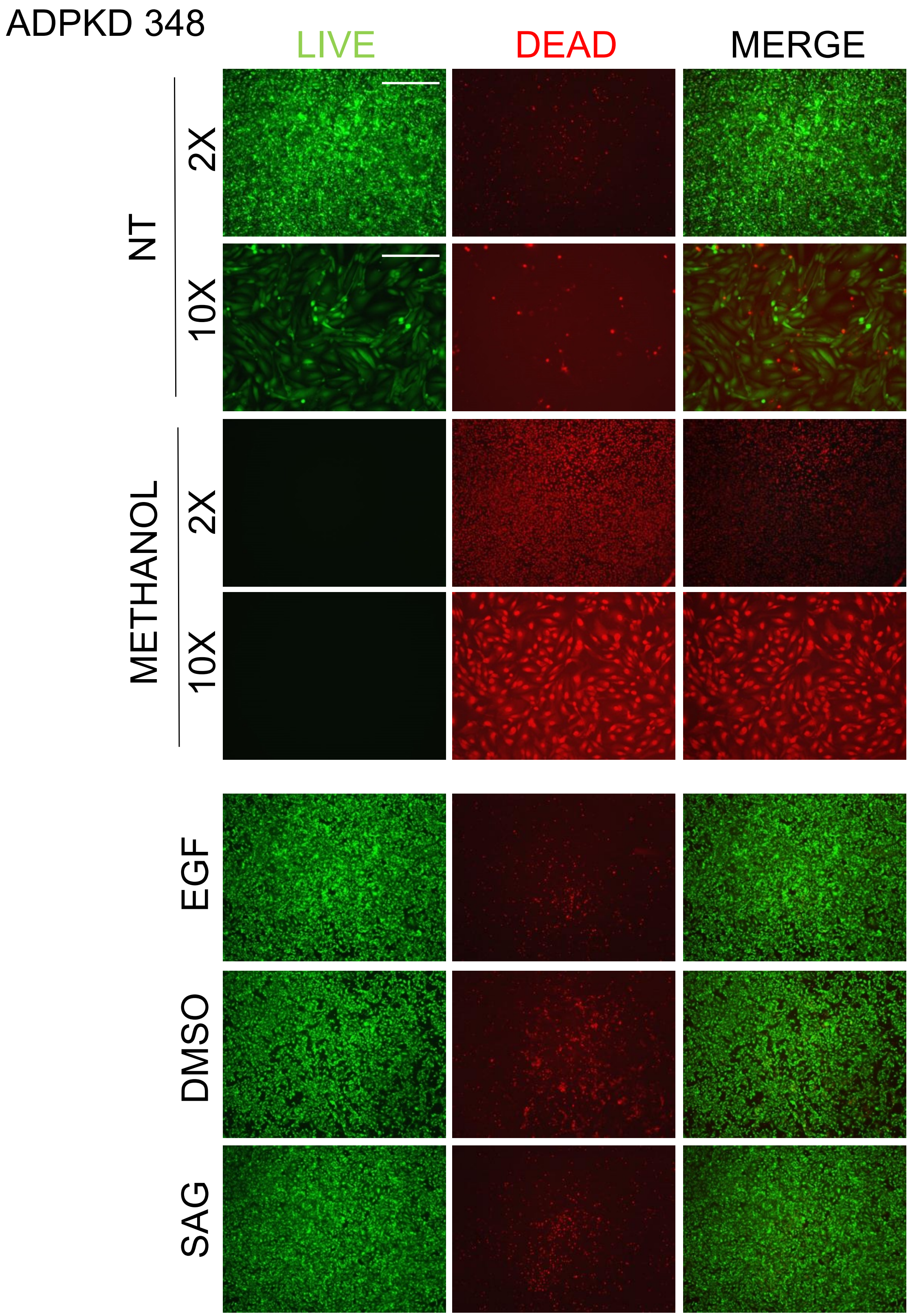

Figure S6. Analysis of cell viability and cytotoxicity of Hh modulators. NHK and ADPKD cells were treated with Hh modulators, alone or together with cAMP, then analyzed for viability/ retention of calcein AM (GFP) in membranes or for non-viability/incorporation of ethidium homodimer-1 (RFP) in nuclei. NT - no treatment. Methanol – 30-minute incubation in 70% methanol was used to cause 100% cell death. Hh treatments were imaged using a 2X objective lens. Scale bar for 2X – 500µm. Scale bar for 10X - 100µm. Assays were performed in 3 NHK and 3 ADPKD cell lines (Summary Table S1).

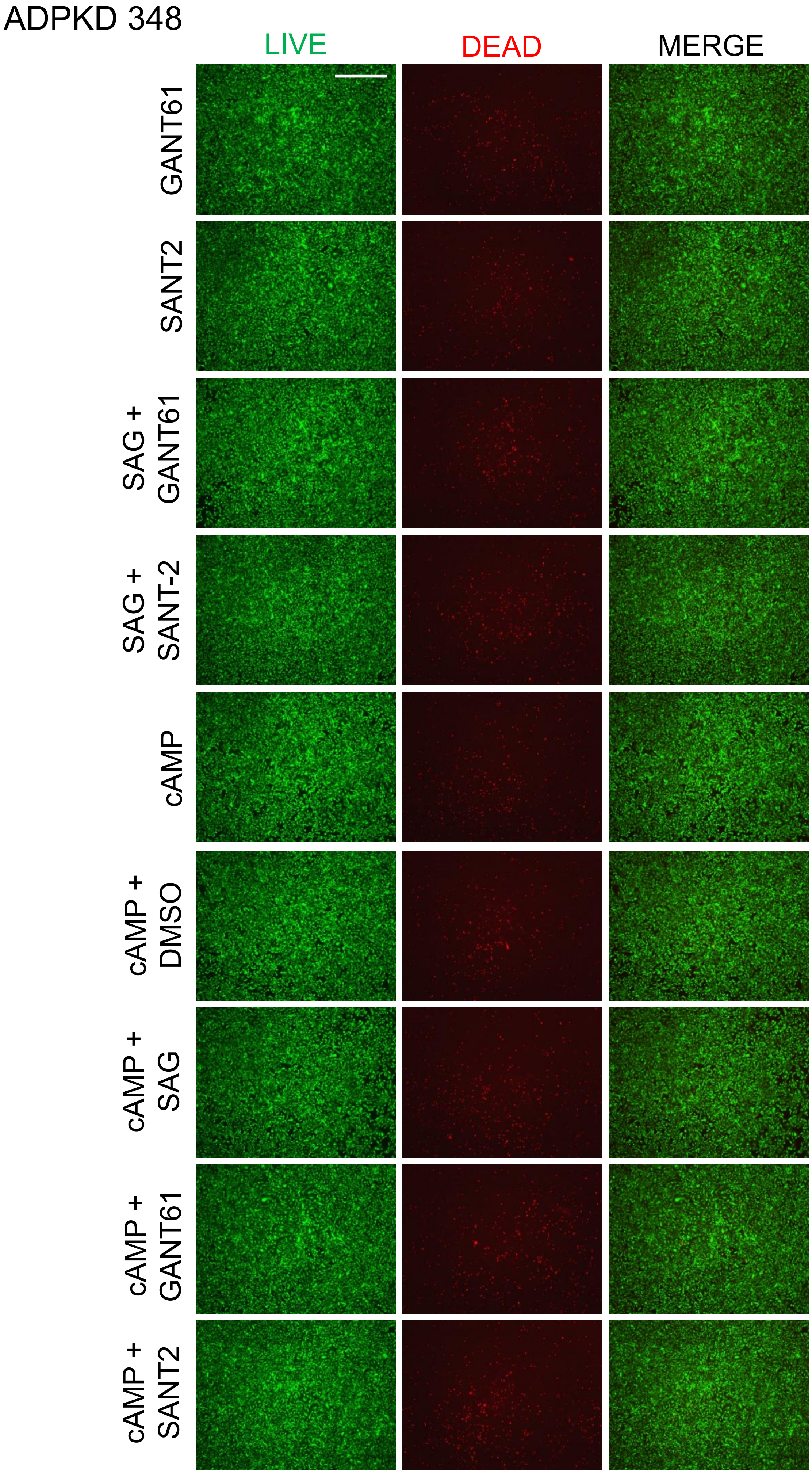

ADPKD 348
LIVE
DEAD
MERGE
GANT61
SANT2
SAG + GANT61
SAG + SANT-2
cAMP
cAMP + DMSO
cAMP + SAG
cAMP + GANT61
cAMP + SANT2

NHK

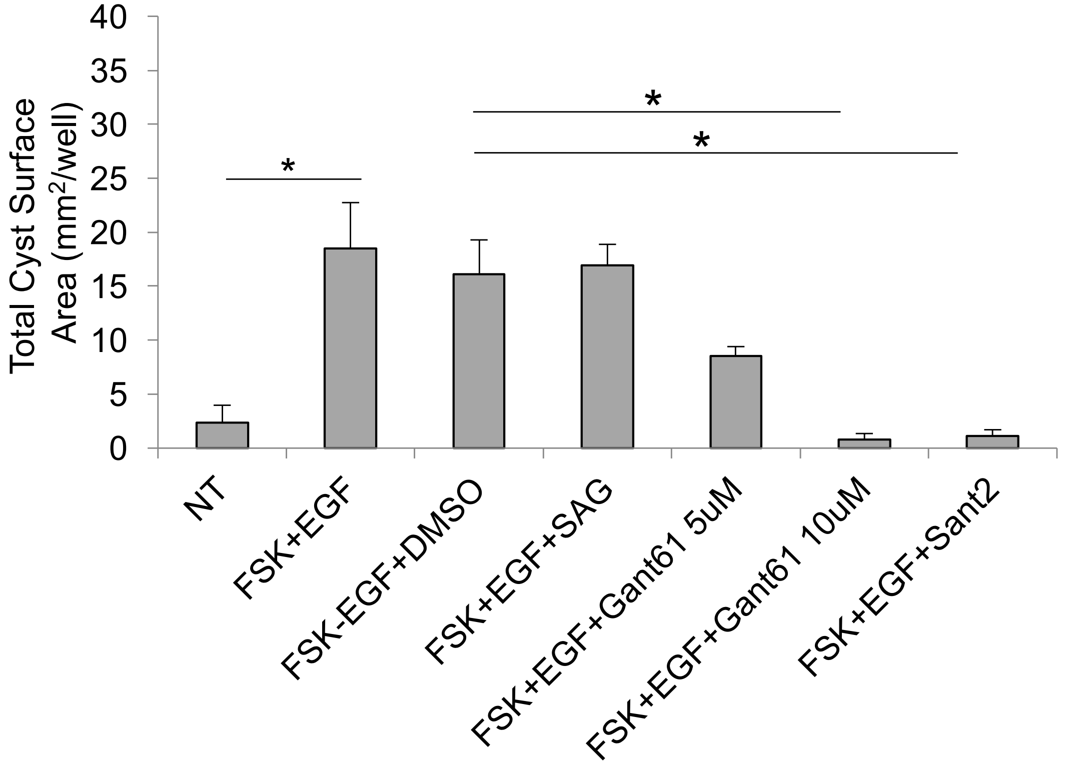

ADPKD

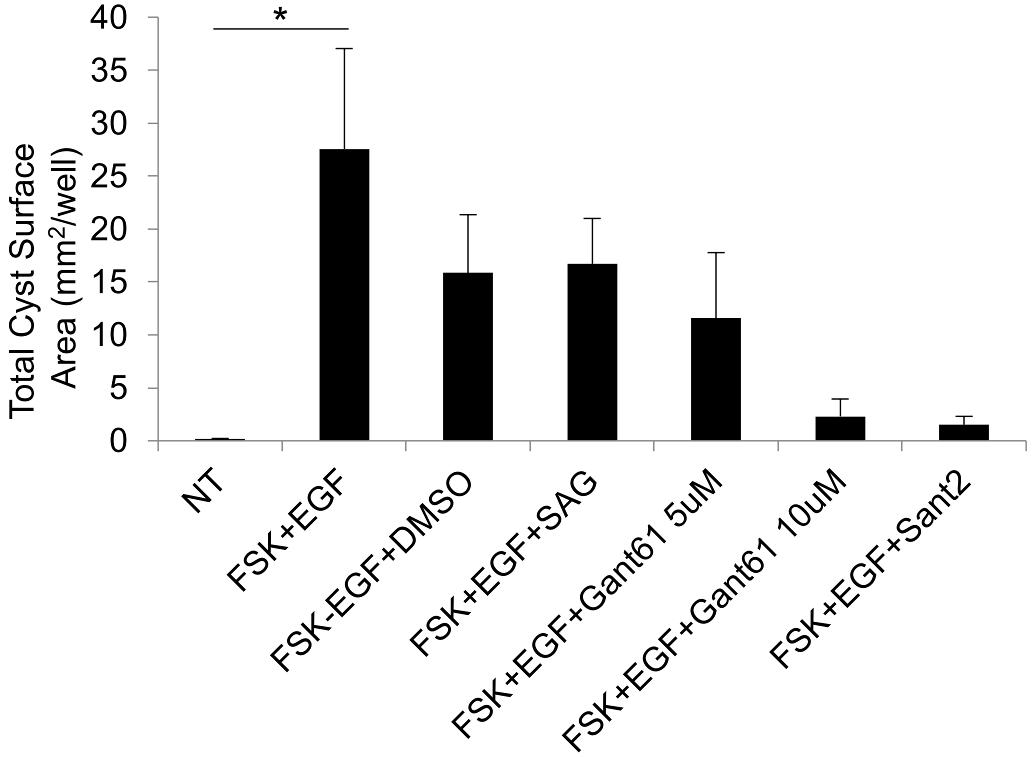

Figure S7. Hh inhibitors reduce FSK- and EGF-induced cyst growth. Quantification of total microcyst surface area per well. FSK-forskolin. Bars represent mean ± SEM of 3 NHK cell lines and 4 ADPKD cell lines. Cells of each line were plated in six replicate wells. Statistical significance was determined by ANOVA and Tukey's test. *$P<0.05$
